# A broadly conserved NERD genetically interacts with the exocyst to affect root growth and cell expansion

**DOI:** 10.1093/jxb/ery162

**Published:** 2018-05-02

**Authors:** Rex A Cole, Valera V Peremyslov, Savannah Van Why, Ibrahim Moussaoui, Ann Ketter, Renee Cool, Matthew Andres Moreno, Zuzana Vejlupkova, Valerian V Dolja, John E Fowler

**Affiliations:** Department of Botany and Plant Pathology and Center for Genome Research and Biocomputing, Oregon State University, Corvallis, OR, USA

**Keywords:** Arabidopsis, cell elongation, cell wall, exocyst, genetic interaction, root development, root hair, root meristem, secretory pathway, tip growth

## Abstract

The exocyst, a conserved, octameric protein complex, helps mediate secretion at the plasma membrane, facilitating specific developmental processes that include control of root meristem size, cell elongation, and tip growth. A genetic screen for second-site enhancers in Arabidopsis identified *NEW ENHANCER of ROOT DWARFISM1* (*NERD1*) as an exocyst interactor. Mutations in *NERD1* combined with weak exocyst mutations in *SEC8* and *EXO70A1* result in a synergistic reduction in root growth. Alone, *nerd1* alleles modestly reduce primary root growth, both by shortening the root meristem and by reducing cell elongation, but also result in a slight increase in root hair length, bulging, and rupture. *NERD1* was identified molecularly as At3g51050, which encodes a transmembrane protein of unknown function that is broadly conserved throughout the Archaeplastida. A functional NERD1–GFP fusion localizes to the Golgi, in a pattern distinct from the plasma membrane-localized exocyst, arguing against a direct NERD1–exocyst interaction. Structural modeling suggests the majority of the protein is positioned in the lumen, in a β-propeller-like structure that has some similarity to proteins that bind polysaccharides. We suggest that NERD1 interacts with the exocyst indirectly, possibly affecting polysaccharides destined for the cell wall, and influencing cell wall characteristics in a developmentally distinct manner.

## Teaser

NERD1, a novel conserved Golgi-localized protein, is linked to the exocyst (a vesicle-tethering complex) by genetic interaction data, and influences growth of the root meristem, elongation zone, and root hairs.

## Introduction

The secretory system in plants is a fundamental determinant of plasma membrane composition and cell wall formation (Luschnig and Vert, 2014; Ebine and Ueda, 2015; Kim and Brandizzi, 2016). Consequently, secretory events drive cell growth and morphogenesis, and influence plant development. For example, selective localization of secretion to specific regions of the cell periphery is essential for polarized growth of pollen tubes, enabling sperm cell delivery and sexual reproduction in flowering plants. Additionally, secretion of substances into the apoplast and delivery of receptors and transporters to the plasma membrane allow for intercellular communication and coordination. Ultimately, the secretory system’s intimate influence on the plasma membrane and extracellular activities facilitates responsiveness and survival within variable abiotic and biotic environments. However, it remains unclear how the secretory process in plants is spatially and temporally regulated to direct particular cargos to specific locations of the plasma membrane, cell wall, or apoplast at the appropriate time. Both the conventional secretory system, i.e. vesicular transport from endoplasmic reticulum to Golgi to plasma membrane, and non-conventional pathways are involved (Drakakaki and Dandekar, 2013; Robinson *et al.*, 2016; van de Meene *et al.*, 2017), but how these pathways are tailored to the dynamic requirements of different cell types is only beginning to be revealed.

The exocyst, an evolutionarily conserved octameric complex, tethers secretory vesicles to specific sites on the plasma membrane prior to exocytosis and modulates secretory activity to achieve an array of specialized functions. In plants, components of the exocyst have been implicated in a range of processes including pollen tube germination and growth (Cole *et al.*, 2005; Hála *et al.*, 2008; Li *et al.*, 2010), cytokinesis (Fendrych *et al.*, 2010; Rybak *et al.*, 2014), secondary cell wall deposition during tracheary element development (Li *et al.*, 2013; Oda *et al.*, 2015), hypocotyl elongation in etiolated seedlings (Hála *et al.*, 2008), determination of meristem size and cell elongation during primary root growth (Cole *et al.*, 2014), Casparian strip formation (Kalmbach *et al.*, 2017), localized disposition of seed coat pectin (Kulich *et al.*, 2010), callose deposition in trichomes (Kulich *et al.*, 2015), and the polar growth of root hairs (Wen *et al.*, 2005; Synek *et al.*, 2006).

The regulation, assembly, and functioning of the exocyst complex in non-plant eukaryotes has been linked to its interactions with small GTPases of the Rho, Ral, and Rab families (Mukherjee *et al.*, 2014), membrane phospholipids (Thapa *et al.*, 2012; Pleskot *et al.*, 2015), plasma membrane scaffolding proteins (Liu and Novick, 2014), and the actin cytoskeleton (Jin *et al.*, 2011; Liu *et al.*, 2012). This interactive milieu helps define exocyst function in yeast and mammals, providing post-translational regulation of key secretory events (Wu and Guo, 2015; Pleskot *et al.*, 2015). In plants, the molecular mechanisms that integrate the exocyst into distinct secretory processes are less well understood. One regulatory mechanism unique to plants is the proliferation and diversification of homologs of the exocyst subunit Exo70 (23 in Arabidopsis), which is hypothesized to allow for specification of particular exocyst functions (Synek *et al.*, 2006; Li *et al.*, 2010; Cvrčková *et al.*, 2012; Vukašinović and Žárský, 2016). In support of this hypothesis, different Exo70 paralogs have been associated with specific cellular processes: Exo70B1 with autophagy (Kulich *et al.*, 2013), Exo70I with arbuscular mycorrhizal symbiosis (Zhang *et al.*, 2015), and Exo70E with the EXPO secretory pathway (Poulsen *et al.*, 2014). Furthermore, in growing pollen tubes, members of the EXO70A, EXO70B and EXO70C subgroups show differential localization patterns and apparent activities (Sekereš *et al.*, 2017; Synek *et al.*, 2017). Other factors that help regulate the exocyst in specific developmental contexts are the scaffolding protein Interactor of Constitutive active ROPs 1 (ICR1) in roots (Lavy *et al.*, 2007); the phosphoinositide PIP2 in pollen tubes (Bloch *et al.*, 2016); ROP2 GTPase (with its effector RIC7) in stomata (Hong *et al.*, 2016); and the combined activities of VETH1–VETH2–COG2 and cortical microtubules in xylem cells (Oda *et al.*, 2015). However, given the breadth of functions known for the plant exocyst, other factors are likely to be involved, including cellular components that interact with the exocyst indirectly, e.g. by enhancing the activity of an exocyst-trafficked protein.

To advance the investigation of exocyst-mediated secretory events in plants, we performed a mutagenesis screen to identify interactors linked to the exocyst’s role in Arabidopsis root growth. In this screen, we identified NEW ENHANCER OF ROOT DWARFISM (NERD1), a protein of unknown function that, based on genetic interaction data, acts with the exocyst to facilitate root and hypocotyl elongation and to influence root hair morphology. *NERD1* is expressed throughout the plant, suggesting a potential role beyond the root and hypocotyl. NERD1 homologs are found throughout the plant kingdom and beyond. Interestingly, the functional interaction of the exocyst with NERD1 is likely to be indirect, and varies dependent on developmental context. We speculate that NERD1 is involved in the modification of cell wall polysaccharides that are important for cell wall expansion and are a cargo for exocyst-mediated transport to the apoplast.

## Materials and methods

### Plant materials and growth conditions

Lines of Landsberg erecta-0 and Columbia-0 ecotype of Arabidopsis with T-DNA insertions were obtained from the SALK Institute (Alonso *et al.*, 2003): *nerd1-2* (At3g51050, SALK 018060C); *nerd1-3* (At3g51050, SALK 051660); *exo70A1-2* (At5g03540, SALK 135462); *sec8-3* (At3g10380, SALK 026204); *sec8-4* (At3g10380, SALK 118129); *sec8-6* (At3g 10380, SALK 091118); and *myo XI-K* (At5g20490, SALK 067972). The *exo84b-1* line was a GABI-Kat line (Rosso *et al.*, 2003; Fendrych *et al.*, 2010). The *EXO84b-GFP* and *GFP-SEC8* lines were previously described (Fendrych *et al.*, 2010). The *nerd1-1* mutant was generated in an ethyl methanesulfonate (EMS) screen that treated ~5000 *sec8-6* seeds with 0.2% EMS for 15 h. M2 generation seed from 4500 M1 plants was collected in pools derived from 16 plant lots. The effectiveness of the mutagenesis was verified by observing greater than 64% of M2 plants with one-quarter aberrant seed, with the gene mutation rate estimated at 1/3000.

Arabidopsis seeds were surface-sterilized, stratified at 4 °C for 3–5 d, and planted on growth medium (1× MS, 2% (w/v) sucrose, and vitamins in 1% (w/v) Bacto-agar) or soil as previously described (Cole *et al.*, 2005). Plants were grown in a climate chamber at 22 °C under long-day conditions (16 h of light per day; 7500 lx), with the exception of those used in hypocotyl elongation experiments. For these, seeds were placed in a lighted incubator at 228C for 2–4 h to stimulate germination and then wrapped in foil, oriented vertically, and placed in a dark box in a 22 °C incubator. After 5 d in the dark, digital images were captured and hypocotyl lengths measured.

To evaluate the effect of Endosidin2, three groups were germinated on MS plates: seedlings that were homozygous for *nerd1-2*; *nerd1-2* siblings complemented by NERD1–green fluorescent protein (GFP); and Col-0 plants. Plants of each genotype were transferred approximately 3 d after germination to plates containing 0, 20, or 40 μM Endosidin2 (ES2), and grown for an additional 4 d before imaging to determine primary root growth rates. DMSO-dissolved ES2 (or DMSO alone as a control, at 0.5% (v/v)) was added to media during plate preparation.

### High-throughput sequencing and analysis

A plant homozygous for *nerd1-1* and lacking a *sec8* allele in a Col-0 background was backcrossed to Ler-0, and the progeny were self-crossed to generate an F2 population. Pooled genomic DNA from 150 F2 plants with the nerd phenotype (i.e. homozygotes) was sequenced via an Illumina HiSeq 2000 to generate 58 million paired-end reads. SHOREmap software (Schneeberger *et al.*, 2009) was used to align the reads to the Arabidopsis genome and assess the frequency of Col (the mutagenized parent) and Ler single nucleotide polymorphisms (SNPs) across the population. All variant SNPs in the ~200 kb region of chromosome 3 harboring *nerd1-1* (Supplementary Fig. S1 at *JXB* online) were then searched against genes to identify candidate mutations with likely deleterious effects.

### Genetic and molecular analyses

DNA extraction from leaves and PCR genotyping for mutants containing T-DNA insertions was performed as previously described (Cole *et al.*, 2005). Primers used in PCR and RT-PCR are shown in Supplementary Table S1. PCR-based genotyping to detect the EMS-generated *nerd1-1* mutation required use of the restriction enzyme *Ava*II after amplification, as a target cleavage site in the At3g51050 genomic sequence was eliminated by the G→A transition in the mutant. To evaluate expression via RT-PCR, roots from approximately 50 10-day-old seedlings of each genotype were harvested from plates and frozen in liquid nitrogen. RNA was extracted from the pooled sample for each genotype using a phenol–chloroform procedure, followed by DNase treatment. First strand cDNA synthesis was performed using Superscript II as per the manufacturer’s specifications (Thermo Fisher Scientific), followed by removal of RNA with RNaseH. The cDNA was used as a template for PCR with primer pairs that amplified the sequence to the 5′ of the mutations, to the 3′ of the mutations, or spanning the sites of the mutations. Primers for *ACTIN2* were included as an internal control.

### Generation of NERD1–GFP and imaging

A 5597-bp-long genomic fragment encompassing the *NERD1* gene along with its putative promoter was PCR amplified from the genomic DNA using KOD Hot Start high-fidelity DNA polymerase (Novagen) and cloned into a modified pMDC32 plasmid using *Sbf*I and *Pac*I sites. Enhanced GFP (EGFP; Clontech) cDNA was added downstream from the *NERD1* open reading frame (ORF) to yield pMDC-NERD1-GFP. The plasmid was mobilized into *Agrobacterium tumefaciens* strain GV3101. Transient expression or coexpression of *NERD1* construct and fluorescent Golgi markers in *Nicotiana benthamiana* leaf epidermal cells was performed by co-infiltrating with *Agrobacterium* strains carrying *NERD1-GFP* and either *STtmd-YFP* or *NAG-mTurq* (Peremyslov *et al.*, 2012) at concentrations equal to 0.2 OD_600_. Imaging was conducted 2 d post-infiltration. Leaf fragments were immersed in water and observed using a Zeiss LSM 780 NLO confocal microscope equipped with a Plan-Apochromat ×63 1.4 NA lens. mTurquoise, GFP, and mCherry were excited with the 405 nm diode laser line, 488-nm argon laser line, or 561-nm He–Ne laser line, respectively. For the simultaneous visualization of two fluorophores, dual channel acquisition of signal for either GFP and mTurquoise or GFP and mCherry was performed sequentially to minimize crosstalk. For Brefeldin A (BFA) sensitivity, Arabidopsis seedlings expressing fluorophore-tagged proteins were treated with 50 μM BFA for 90 min, and the BFA-sensitive endomembrane compartments were imaged in root epidermal cells.

Evaluation of cortical cell files and root growth parameters in *nerd1* mutants using confocal microscopy was performed as previously described (Cole *et al.*, 2014). Briefly, images of roots grown on vertical plates were captured on day 5 and day 7 after germination to determine root growth rates. The 7-day-old seedlings were stained with propidium iodine and then imaged with a Zeiss LSM 780 NLO confocal microscope system. Multiple digital images were taken to capture two cortical cell files for each root, one on each side of the longitudinal midline from the quiescent center to the differentiation zone. Cell widths and lengths were measured. The cortical cell length profile combined with the root growth rate allowed estimations of the number of cells in the meristem, the cell production rate, and the length of the cell cycle for each root. Measurements (e.g. root lengths, hypocotyl lengths, root hair dimensions, and root cortical cell lengths and widths) from confocal digital images were achieved using ImagePro analysis software (MediaCybernetics). Transmitted light images of hypocotyl and root hair specimens were captured with a Leica DFC 295 digital camera attached to a Zeiss Stemi SV 11 dissecting microscope, utilizing Leica Application Suite v3.8.

## Results

### Screening for exocyst interactors

Mutations in genes encoding components of the exocyst complex in Arabidopsis result in root growth defects that vary from a mild decrease in growth rate in some mutants (e.g. *sec8-6* and *exo70A1*) to severe dwarfism in others (e.g. *exo84b-1* and *sec8-3*) (Cole *et al.*, 2014). Additionally, mutations of some exocyst components reduce the length of root hairs (Synek *et al.*, 2006). We reasoned that a protein that interacts with the exocyst could be revealed if its mutation accentuated the root growth defect of an exocyst mutation that by itself results in only a mild phenotype. Therefore, we screened an EMS-treated population of seedlings homozygous for the mild *sec8-6* mutation in a Col-0 background to identify such second-site enhancers.

**Fig. 1.**
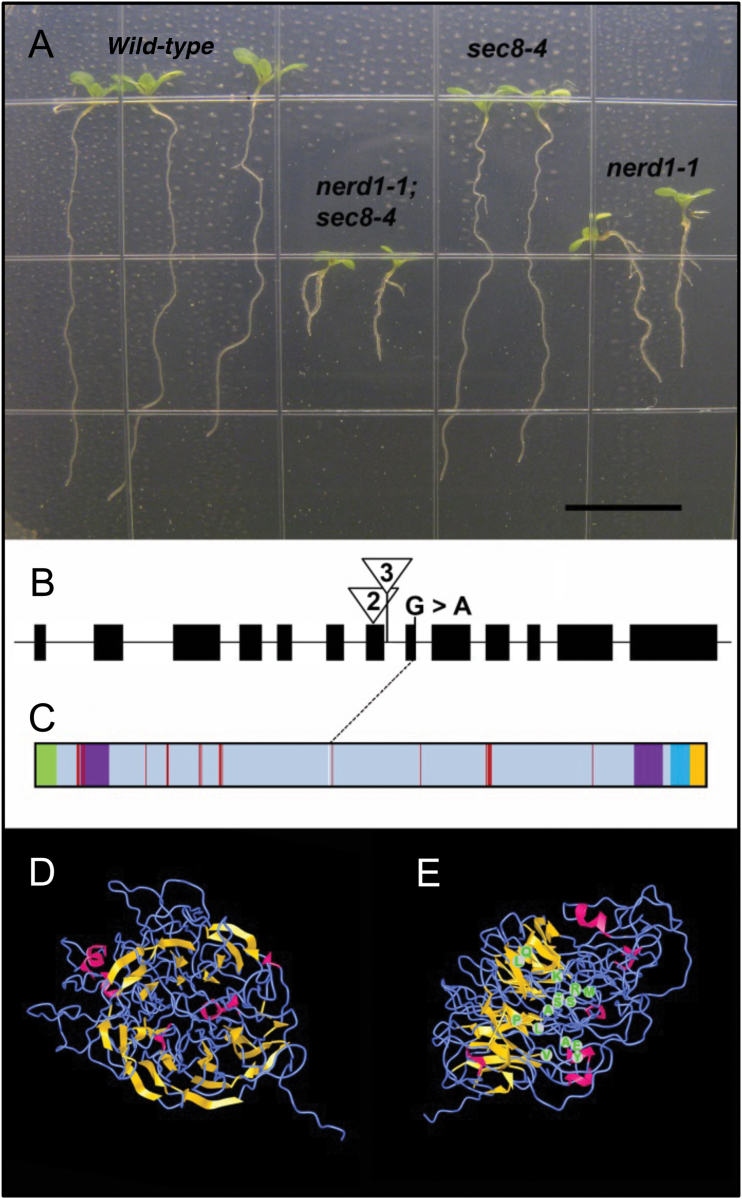
*NERD1* encodes a transmembrane domain protein important for wild-type root development. (A) The combination of *nerd1-1* and *sec8-4* mutations results in a more severe root growth defect than either single mutation. Scale bar: 1 cm. (B) Map showing exons of *NERD1* (At3g51050), the site of the G→A point mutation at a splice junction in *nerd1-1*, and the sites of the T-DNA insertions *nerd1-2* (triangle 2: SALK_018060) and *nerd1-3* (triangle 3: SALK_051660). (C) Schematic representation of NERD1 primary sequence features, showing an N-terminal signaling peptide (green), FG–GAP domains (purple), transmembrane domain (aqua), cytoplasmic domain (yellow), and residues predicted to form a calcium-binding pocket by RaptorX (red). The blue diagonal line between (B) and (C) shows where the splice site mutation in *nerd1-1* is predicted to affect the NERD1 polypeptide. (D) NERD1 tertiary structure predicted by RaptorX, showing a β-propeller in yellow and α-helices in magenta. (E) Side view of the model in (D) with locations of residues in the predicted calcium-binding pocket (green).

Plants from M2 pools exhibiting both short roots and aberrant root hairs—dubbed the new enhancer of root dwarfism (nerd) phenotype—were outcrossed to a wild-type line, self-pollinated, and screened again for the phenotype in ~1/16 of the progeny, as expected for second site enhancers. After screening 45 pools, we recovered exactly one such mutation, *nerd1-1*, which, when combined with *sec8-6*, leads to profound dwarfism throughout the plant and shorter primary roots (~25% of wild-type length, with some demonstrating terminated growth). In addition, root hairs in *nerd1 sec8-6* double mutants are occasionally misshapen (Supplementary Fig. S2). To verify that the genetic interaction was not specific to a particular *SEC8* allele, the *nerd1-1* mutation (isolated after a series of backcrosses to Col-0) was combined with another mild allele, *sec8-4*, yielding a similar result (Fig. 1A). Intriguingly, initial observations indicated that *nerd1* plants, in the absence of a *sec8-6* or *sec8-4* mutation, have a similar, but less severe phenotype—e.g. root length ~75% of wild-type, and less frequently misshapen root hairs.

### Molecular identification of *NERD1*

To identify the *nerd1-1* lesion, we used high-throughput sequencing of a pooled population of mutant plants to identify a ~200 kb region of chromosome 3 tightly linked to *nerd1* (Supplementary Fig. S1). This region encompassed ~65 protein-coding genes, only two of which harbored putative EMS-generated G→A mutational differences from the Col-0 reference sequence linked to *nerd1-1*. Both mutations were validated via Sanger sequencing. The best candidate for *nerd1-1* appeared to be a change in a conserved splice acceptor site at the ninth exon of At3g51050 (Fig. 1B). To confirm the molecular identity of *NERD1*, two independent T-DNA insertion alleles in At3g51050 (Fig. 1B) were obtained from the Salk mutant collection, both of which were associated with short root and root hair defects. Subsequent complementation tests between heterozygotes for all three alleles showed the *nerd1* root growth and root hair phenotype appearing in approximately 25% of the progeny, verifying that the two insertion alleles (designated *nerd1-2* and *-3*) were indeed inactivating this same locus affected in the original *nerd1-1* line, and proving that At3g51050 corresponds to the *NERD1* gene. This segregation pattern further shows that *nerd1* mutants do not have a significant gametophyte-derived transmission defect. Notably, pollen is the developmental stage, across 105 stages assessed in the Genevestigator database (Grennan, 2006), associated with lowest expression of At3g51050. The absence of a significant transmission defect in *nerd1* mutants, and the low level of expression, suggests that *NERD1* function is not as central to polarized growth in pollen tubes as it is in root hairs.

In addition to the similar phenotypic severity of each of the three alleles, RT-PCR assays suggested that all three were nulls, each generating aberrant transcripts that likely produce non-functional protein. NERD1 transcripts in *nerd1-1* homozygotes were shorter than wild-type in the region spanning the point mutation, and thus were likely mis-spliced, whereas transcripts in *nerd1-2* and *-3* were detected upstream, but not downstream from their respective T-DNA insertions (Supplementary Fig. S3).

The 698 amino acid-long NERD1 sequence is broadly conserved in plants, and homologs are detectable in non-plant species (Supplementary Dataset S1). The Gramene EnsemblPlants database (Kersey *et al.*, 2016) identifies *NERD1* homologs in 42 Viridiplantae species, primarily angiosperms, but also including more distantly related Archaeplastida species, including members of Bryophyta (*Physcomitrella patens*), Lycopodiophyta (*Selaginella moellendorffii*), and Chlorophyta (*Chlamydomonas reinhardtii* and *Ostreococcus lucimarinus*). In Arabidopsis and 26 other Viridiplantae species, *NERD1* is identified as a single copy gene. Certain regions of NERD1 also show notable similarity to proteins in both Metazoan and Amoebozoan species (Supplmentary Dataset S1).

Protein modeling software was used to predict potential structural characteristics of NERD1 (Fig. 1C; Supplementary Table S2; Supplementary Figs S4 and S5; Supplmentary Datasets S2 and S3). Arabidopsis NERD1 contains an N-terminal signaling peptide that is well conserved across all plant species and is indicative of association with the secretory system (TargetP 1.1, Emanuelsson *et al.*, 2000). A single-pass transmembrane domain is identified near the C-terminus of the protein, leaving a short 18–23 amino acid cytoplasmic tail. Homology threading programs (3DLigandSite, Wass *et al.*, 2010; Raptor-X, Källberg *et al.*, 2012; SWISS-Model, Biasini *et al.*, 2014; Phyre2, Kelley *et al.*, 2015) that compared NERD1’s primary and secondary structure with proteins with tertiary structures solved by x-ray crystallography (Supplmentary Dataset S2) predict (with 90–98% confidence) that the non-cytoplasmic portion of NERD1 folds into a globular protein with a β-propeller structure: seven predicted β-sheets arranged radially and pseudosymmetrically around a central axis (Fig. 1D–E; Supplementary Fig. S5). β-Propellers are widely used as structural scaffold, providing a surface for ligand binding and enzymatic activity (Kopec and Lupas, 2013). In addition, NERD1’s β-propeller contains a putative calcium-binding pocket. The predicted tertiary structure resembles templates for some pyrroloquinoline quinone-dependent enzymes (e.g. alcohol dehydrogenases) and, intriguingly, shares similarity to proteins that interact with polysaccharides or glycoproteins, including lectins, integrins, carbohydrate binding proteins, and perhaps most notably, some pectin lyases and xyloglucanases (Supplmentary Dataset S2). Overall, however, the full length of NERD1 is not strictly homologous to members of any known protein family. Although the exact 3D structure and molecular function of NERD1 remain uncertain, these predictions raise the intriguing possibility that NERD1 is an integral membrane protein that interacts with polysaccharides, potentially in a calcium-dependent manner. Notably, NERD1 (At3g51050) mRNA is expressed throughout most of the Arabidopsis plant, with little change induced by developmental or environmental variables (Grennan, 2006), suggesting that NERD1 is a component of most plant cells.

### Localization of NERD1

**Fig. 2.**
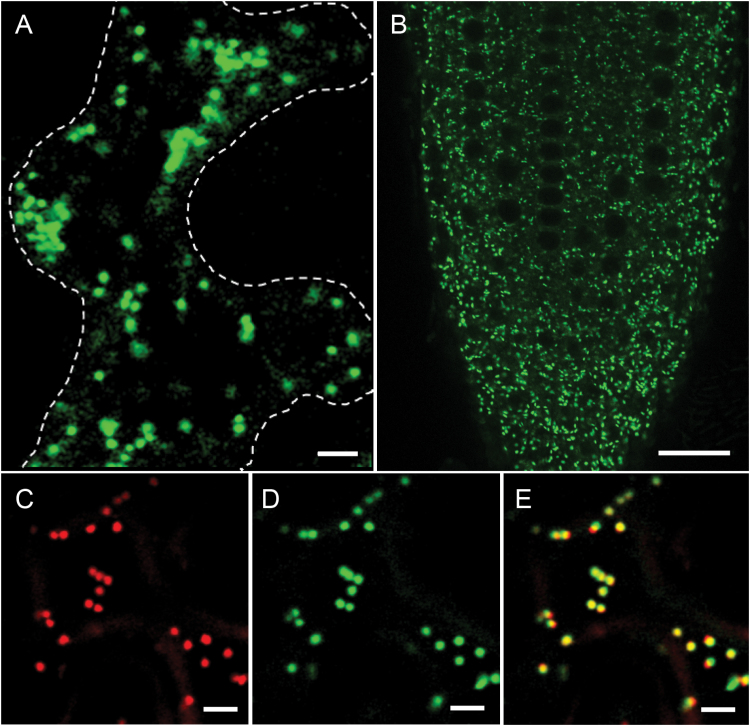
NERD1–GFP localizes to the Golgi. (A) NERD1–GFP in *Nicotiana benthamiana* leaf cells. (B) Root tip of a *NERD1-GFP*-complemented *nerd1-2* mutant. (C–E) Co-localization of NERD1 with the Golgi marker: (C) STtmd::mCherry (sialyltransferase transmembrane domain), (D) NERD1–GFP, and (E) merged image. Scale bars: 5 μm (A, C, D, E) and 20 μm (B).

Previous large-scale proteomic analyses detect NERD1 at the plasma membrane and/or Golgi (Mitra *et al.*, 2009; Zhang and Peck, 2011; Parsons *et al.*, 2012; Heard *et al.*, 2015). To validate and refine these findings, a genomic clone harboring the native promoter and complete ORF of NERD1 was tagged with GFP at the 3′ end and expressed transiently in *Nicotiana benthamiana* or used to generate transgenic Arabidopsis plants. Confocal microscopy of leaf epidermis cells of *N. benthamiana* revealed that the tagged protein is present in small motile bodies of *ca* 1 μm in diameter that resembled Golgi stacks (Fig. 2A). To investigate the nature of these bodies, we examined cells of *N. benthamiana* co-expressing NERD1–GFP and one of two different Golgi markers: STtmd–Cherry or an *N*-acetylglucosaminyl transferase fused to fluorescent protein mTurquoise (NAG–mTurq) (Peremyslov *et al.*, 2012). In all cells examined, the GFP signal co-localized with these Golgi markers, indicating that NERD1 is primarily present in Golgi (Fig. 2C–E; Supplementary Fig. S6).

The functionality of the NERD1–GFP fusion protein was validated by genetic complementation. To this end, the NERD1–GFP expression cassette was stably transformed into an Arabidopsis *nerd1-2* heterozygote line, which was subsequently self-crossed. A progeny plant homozygous for the *nerd1-2* mutation but phenotypically wild-type was identified and self-crossed. PCR genotyping verified that all the resultant seedlings were homozygous for the *nerd1-2* mutation. One-fourth of these plants (13 of 52) exhibited the nerd root phenotype (shorter roots: 7.6 ± 1.3 mm *versus* wild-type 13.0 ± 2.6 mm, *t*-test *P*<10^–12^; and altered root-hair morphology); all plants exhibiting the nerd phenotype were negative for the cassette presence and NERD1–GFP expression. In contrast, the fusion cassette was present and expressed in all the seedlings that were phenotypically wild-type, indicating that it provides a functional NERD1 protein. Confocal microscopy revealed that the NERD1–GFP in a *nerd1-2* mutant background was localized to mobile punctate structures in the cytoplasm (Fig. 2B), similar to the observations in *N. benthamiana*, and consistent with NERD1 localization in the Golgi. To further validate association of NERD1–GFP with Golgi, we investigated sensitivity of the fluorescent bodies to BFA, which disrupts Golgi architecture and induces formation of an endoplasmic reticulum (ER)–Golgi hybrid compartment (Ritzenthaler *et al*, 2002). Arabidopsis seedlings stably expressing either NERD1–GFP or, as a control, NAG–mTurq, were incubated with this drug. As expected, BFA treatment resulted in formation of a typical BFA compartment marked by either NAG–mTurq or NERD1–GFP in each line (Supplementary Fig. S7), strongly supporting the Golgi residence of NERD1–GFP.

**Fig. 3.**
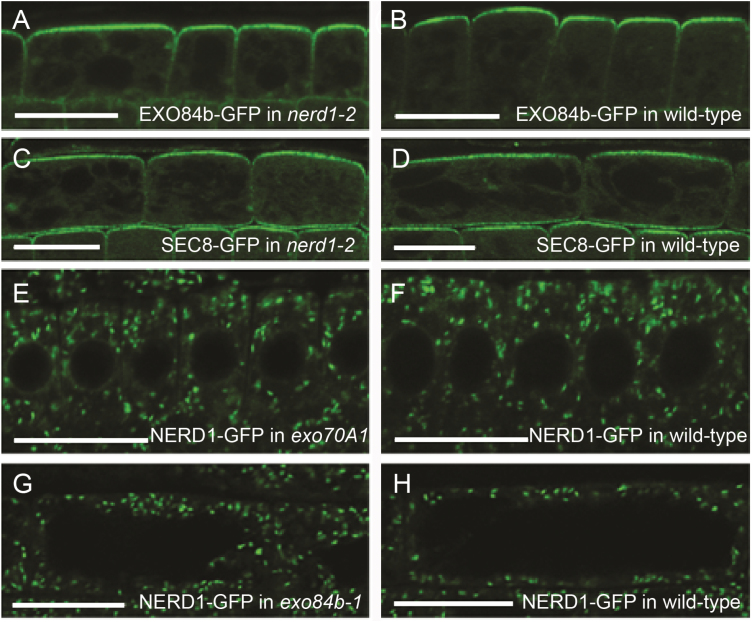
Subcellular localizations of NERD1 and exocyst markers are independent of each other. The localization of exocyst markers to the outer surface of root epidermal cells of *nerd1-2* mutants (A, C) is similar to that in wild-type siblings (B, D). Conversely, the predominant localization of NERD1–GFP in the cytoplasm of root epidermal cells of exocyst mutants (E, G) is similar to that of wild-type siblings (F, H). Shown are epidermal cells in the root transition zone (A–D, G, H) and meristem (E, F). Confocal images provide radial longitudinal sections through the center of the root (A, B, E, F) in which the upper portion of cells shown are on the root surface. In tangential sections (C, D, G, H) parallel with the root surface the lateral walls of the epidermal cells are shown. Scale bars: 20 µm. (This figure is available in colour at *JXB* online.)

The localization of NERD1–GFP in the Golgi is notably distinct from that observed for components of the exocyst at the plasma membrane and in the cytoplasm (Fendrych *et al.*, 2010, 2013; Li *et al.*, 2013; Oda *et al.*, 2015). This suggests that the interaction between NERD1 and the exocyst is not a direct interaction at the plasma membrane, as we had initially hypothesized. One possibility is that NERD1 in the Golgi is important for correct transit of exocyst components to the plasma membrane. Thus, we tested whether the *nerd1* mutation causes a mislocalization of the exocyst by imaging GFP-labeled exocyst components. Both EXO84–GFP and SEC8–GFP localization patterns at the plasma membrane in *nerd1* mutant roots were indistinguishable from their localization in wild-type controls (Fig. 3A and B, and 3C and D, respectively). Thus, the *nerd1* mutant root phenotype is not explained by a mislocalization of the exocyst. The converse was also considered, i.e. do exocyst mutations result in altered localization of NERD1? Observation of NERD1–GFP in the roots of *exo70A1* and *exo84b* mutants revealed that NERD1 localization (i.e. in punctate structures within the cytoplasm) was not altered by mutation of these exocyst components (Fig. 3E–H). These data further argue that the genetic interaction of *NERD1* and exocyst mutants is indirect.

### Genetic interactions with *NERD1* mutants depends on developmental context

The apparent absence of co-localization of NERD1 and the exocyst motivated a quantitative assessment of their phenotypes and genetic interactions in three distinct developmental contexts. Mutations of *NERD1* combined with mutations of exocyst components were examined for their effects on primary root growth, cell elongation in etiolated hypocotyls, and the polarized growth of root hairs. Intriguingly, while all three developmental contexts involve some degree of cell expansion and are impacted by both NERD1 and the exocyst, as detailed below, the specific genetic interactions varied, depending upon context.

### Growth of primary root and etiolated hypocotyl

**Fig. 4.**
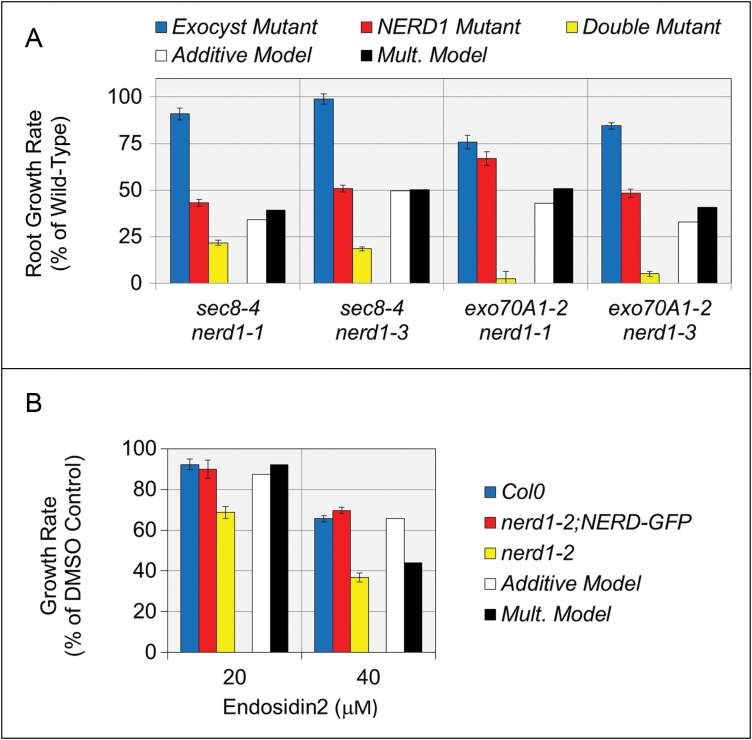
NERD1 acts synergistically with exocyst components to affect primary root growth. (A) Mutation of exocyst components *sec8-4* or *exo70A1-2* results in a mild reduction in primary root growth rate compared with wild-type (blue bars), while mutation of *nerd1* (*nerd1-1* or *nerd1-3*, red bars) results in an approximately 50% reduction in growth rate. The combination of a mutation in an exocyst component with a *nerd1* mutation (green bars) results in a severe root growth defect that is more severe than predicted by additive (*P*<0.001, *z*-test) or multiplicative models (*P*<0.0001, *z*-test), indicating a synergistic interaction. (Error bars: SE; *n*=19–67 roots for each genotype.) (B) Compared with DMSO-treated controls, root growth was significantly more reduced in *nerd1-2* mutants when 20 or 40 μM Endosidin2, an exocyst inhibitor, was added to the growth medium, compared with either Col-0 or *nerd1-2; NERD-GFP* complemented seedlings. At both concentrations, the reduced growth rate in the *nerd1-2* mutant treated with Endosidin2 was more than would be predicted by additive or multiplicative models (*P*<0.001, *z*-test). (Error bars: SE; *n*=13–17 roots for each genotype/Endosidin2 concentration.)

Primary root growth was examined in plants harboring the *nerd1-1* or *nerd1-3* mutation in combination with a mutation in an exocyst component, *exo70A1* or *sec8-4*. A comparison of sibling plants confirmed that the primary root growth defect was more severe in the double mutants than in either of the single mutants (Fig. 4A). Additive and multiplicative models were used to predict the severity of the root growth defect that would be observed if the mutations were non-interacting (Hála *et al.*, 2008; Mani *et al.*, 2008). The observed growth rate defect in the double mutants was much more severe than predicted by either model, verifying a synergistic interaction between NERD1 and exocyst in root growth. To further substantiate the functional interaction between NERD1 and the exocyst, *nerd1-2* mutants were treated with the chemical Endosidin2 (ES2), which inhibits exocyst function by interacting with EXO70A1 (Zhang *et al.*, 2016). As expected, root growth rates of control *nerd1-2* plants harboring the NERD1–GFP construct were not significantly different from those of Col-0 seedlings grown on media containing 0, 20, or 40 μM ES2 (Fig. 4B). In contrast, *nerd1-2* homozygotes are significantly more sensitive to the effect of ES2 on root growth rate, at both 20 and 40 μM ES2. Thus, similar to the genetic interaction, the effect of pharmacological inhibition of the exocyst on root growth in *nerd1-2* mutants is more than would be predicted by multiplicative or additive models (Fig. 4B).

The primary root growth defect in exocyst mutants is due to both a reduced number of cells dividing in a shorter meristem, and a slower rate of cell expansion in the elongation zone (outside the meristem) (Cole *et al.*, 2014). Given the functional interaction between NERD1 and the exocyst in the primary root, we were curious to know if *nerd1* mutant effects could be attributed to one or both of these underlying mechanisms. Consequently, cortical cell files in the root tips of *nerd1* mutants were examined by confocal microscopy and compared with those in the root tips of Col-0 and *exo84b* (an exocyst mutant with a severe root growth defect) grown on the same vertical plates (Table 1). Similar to exocyst mutants with severe root growth defects (e.g. *exo84b-1*) (Cole *et al.*, 2014), the reduced primary root growth in *nerd1* mutants arises from both less cell elongation, leading to shorter mature cells, and a reduced number of cells dividing in shorter meristems. These defects were quantitatively similar for all three *nerd1* alleles, and less severe than in *exo84b-1*. Notably, mature cortical cell widths in *nerd1* mutants are similar to those in Col-0, indicating that the *nerd1* defect is specific to cell elongation. This contrasts with exocyst mutant cells, in which overall mature cortical cell size, both length and width, is reduced. Thus, NERD1 appears to be more specifically involved in longitudinal expansion of lateral walls in the root elongation zone.

**Table 1.** Primary root growth parameters of *nerd1* mutants compared with Col-0 and *exo84b-1*

Genotype	Roots evaluated	Root growth(μm h^−1^)		Meristem size (no. of cells)		Mature cell length (μm)			Mature cell width (μm)			Cell production (cells h^−1^)		Estimated length of cell cycle (h)	
		Mean	SD	Mean	SD	*n* ^*a*^	Mean	SD	*n* ^*a*^	Mean	SD	Mean	SD	Mean	SD
Col-0	9	469.3	43.8	49.6	4.4	264	200.9	43.0	84	28.0	2.57	2.33	0.24	14.7	1.5
*exo84b-1*	10	51.2	6.6	10.0	1.9	278	86.6	21.8	84	13.2	1.53	0.59	0.08	11.4	1.1
*nerd1-1*	6	248.1	30.9	**33.8**	4.0	82	**144.0**	30.1	89	*27.0*	3.98	**1.77**	0.23	13.3	1.5
*nerd1-2*	6	231.2	38.8	**32.4**	3.0	112	**153.3**	38.5	87	*28.4*	3.34	**1.50**	0.17	14.5	0.8
*nerd1-2*	6	262.7	21.6	**35.3**	0.9	89	**130.7**	36.5	75	**29.9**	3.81	*2.08*	0.41	12.0	2.3

Values highlighted in bold are significantly different from Col-0 and *exo84b-1* (*P*<0.001, *t*-test). Values highlighted in italic are significantly different from *exo84b-1*, but not Col-0 (*P*<0.001, *t*-test).

^*a*^ *n*=number of cells measured.

Elongation of the hypocotyl in etiolated seedlings, in contrast to the more complex process of root growth, is due solely to cell elongation, thereby providing a second and more specific system to evaluate the genetic interaction of *NERD1* and exocyst mutants in cell elongation. Hypocotyl lengths and epidermal cell lengths in the hypocotyls of 5-day-old dark grown *nerd1-3 sec8-4* double mutant seedlings were evaluated and compared with similar measurements in single mutant and wild-type siblings (Supplementary Fig. S8). As in roots, *nerd1-3* and *sec8-4* interact synergistically to reduce hypocotyl lengthening (Supplementary Fig. S8B). Furthermore, the effect on the hypocotyl was associated with a synergistic defect in cell elongation (Supplementary Fig. S8C), again similar to the results in the primary root. These data are consistent with expression data pointing to a role for NERD1 and the exocyst in cell growth throughout the plant.

### Polarized growth of root hairs

**Fig. 5.**
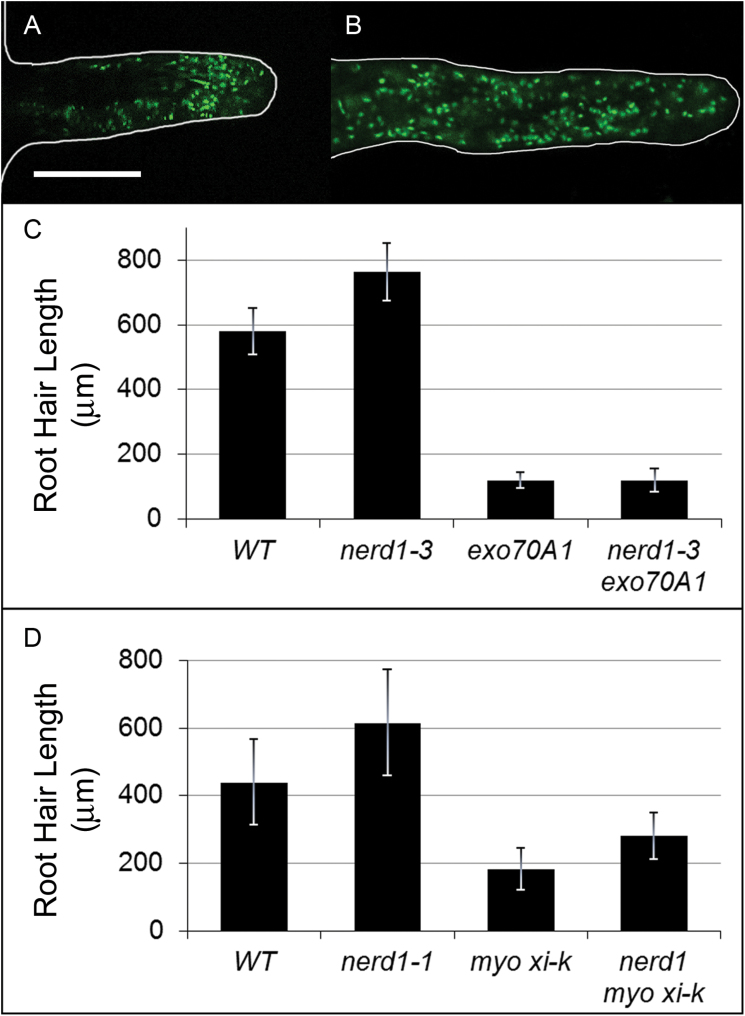
NERD1 is epistatic to the exocyst in the root hair. Root hair lengths were examined for plants growing on vertical plates so that their roots were on the surface of the growth medium, i.e. at the medium–air interface. NERD1–GFP is localized to mobile punctate structures in both wild-type (A) and *exo70A1* (B) root hairs (scale bars for A and B: 20 μm). Mature root hairs of *nerd1-3* and *nerd1-1* mutants are longer than for wild-type siblings (*P*<0.0001, *t*-test). (C) Extremely short root hair lengths are observed in *exo70A1* mutants compared with wild-type (*P*<0.0001), and this phenotype is not altered by the addition of a *nerd1-3* mutation in the double mutant (*P*=0.3). (*n*=190 for WT, *nerd1-3*, and *exo70A1*, *n* =51 for double mutant.) (D) The *myo xi-k* mutant has short root hairs compared with wild-type (*P*<0.0001). In *nerd1-1; myo xi-k* double mutants, root hairs are longer than those of *myo xi-k* (*P*<<0.0001), but still far shorter than wild-type (*P*<0.001). (*n*>164 for WT, *nerd1-1* and *myo xi-k*; *n*=79 for double mutant; error bars: SD.) (This figure is available in colour at *JXB* online.)

One phenotype leading to selection of the initial *nerd1* allele was altered root hair morphology. Short root hairs are characteristic of several exocyst mutants (e.g. *exo70A1* and *exo84b*) (Synek *et al.*, 2006), whereas root hairs that are wild-type in length are observed in other exocyst mutants (e.g. *sec8-4*). As in the primary root, abrogating exocyst function does not alter NERD1 localization patterns in the root hair (Fig. 5A, B). Nevertheless, comparison of root hairs in *exo70A1 nerd1-3* double mutants and sibling single mutants revealed a genetic interaction (Fig. 5C). Surprisingly, the average root hair length in the single *nerd1* mutants was significantly longer than that of their wild-type siblings, indicating that NERD1 limits cell growth in this context. As a second surprise, in contrast to the synergistic interaction in primary root growth, *exo70A1 nerd1-3* double mutants have short root hairs of similar size to those of *exo70A1* single mutants. That is, the effect of the *nerd1-3* mutation in increasing average root hair length is masked (epistasis), suggesting that, in root hairs, the exocyst is required for manifestation of NERD1’s root hair length limiting activity. To determine whether the epistatic interaction of NERD1 and the exocyst was specific, we tested for interactions with another mutation that affects the secretory pathway in root hairs, *myosin xi-k*. Mutation of *myosin xi-k* results in shorter root hairs, likely due to inhibition of cytoplasmic streaming that drives secretory vesicle transport (Peremyslov *et al.*, 2008; Peremyslov *et al.*, 2012; Park and Nebenführ, 2013; Peremyslov *et al.*, 2015). The double *nerd1-1 myo xi-k* mutant demonstrates an additive phenotype: root hairs are longer than with the *myo xi-k* mutation alone, but not as long as wild-type (Fig. 5D). Thus, the epistatic interaction of *nerd1* and *exo70A1* mutants is specific, further arguing for a close functional relationship between NERD1 and the exocyst. Moreover, the differing outcomes of the interaction in root hairs versus primary roots (epistatic versus synergistic, respectively) argue that this relationship depends on cellular and developmental context.

**Fig. 6.**
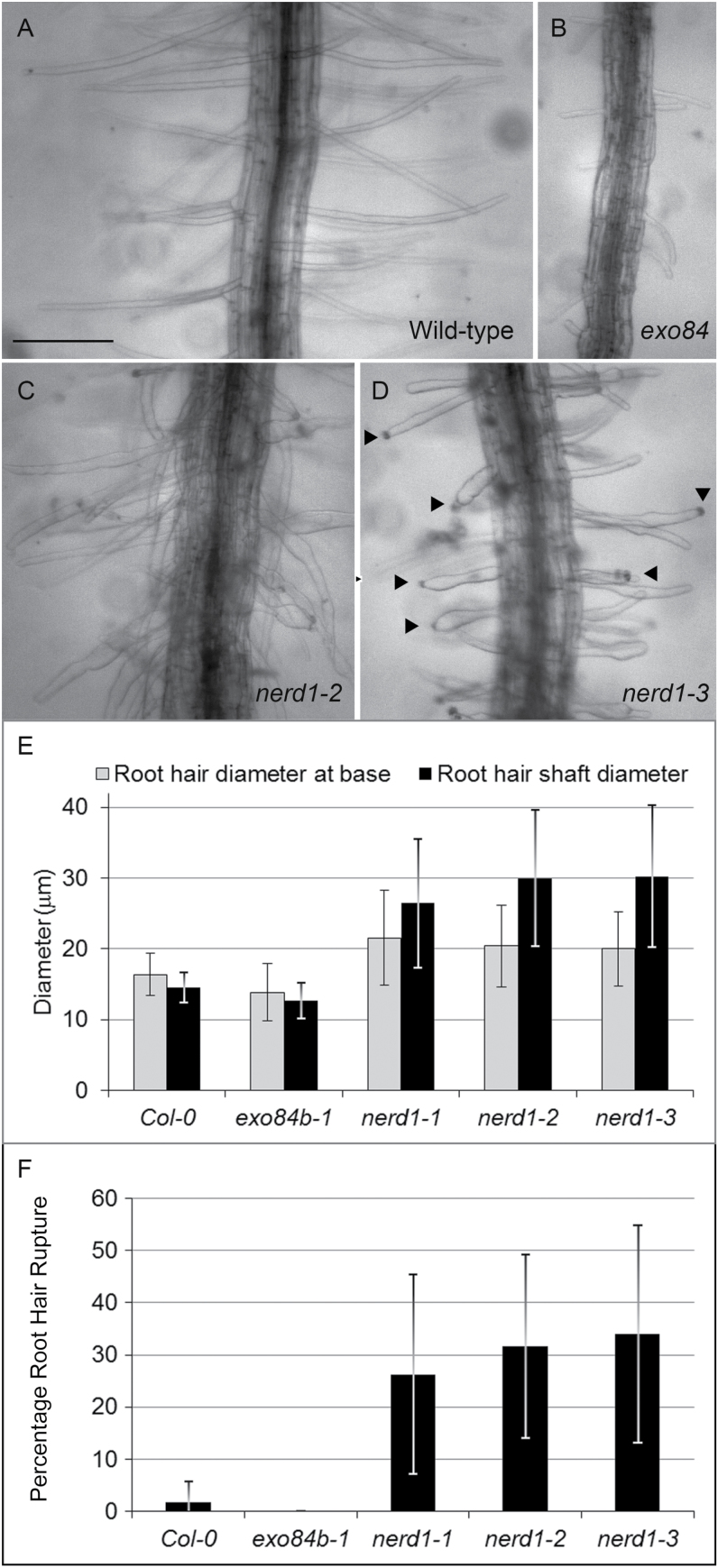
NERD1 affects morphology of root hairs growing within agar medium. (A) Col-0 (wild-type), (B) *exo84b-1*, (C) *nerd1-2*, (D) *nerd1-3*. Arrowheads: ruptured root hair tips; scale bar: 200 µm. (E, F) Root hairs of *nerd1* mutants (*n*=~200 root hairs for each allele) were significantly wider at the base and at the widest part of their shaft than either Col-0 (*n*=203) or *exo84b-1* (*n*=79) (E), and showed a significantly higher incidence of root hair rupture (F). (Root hairs measured from 18 roots for each genotype, *P*<0.001, *t*-test; error bars: SD.)

Additional insight into the role of NERD1 in root hair growth was gained by a closer examination of the morphology of root hairs in *nerd1* mutants, which exhibit branches, inflated bases, or bulbous shapes, morphologies that are rare in wild-type siblings (Supplementary Fig. S2). These deviant morphologies are more consistently observed in roots that are growing within agar medium, rather than on the agar surface (where root hairs predominantly extend into the air). Consequently, root hair morphology within the medium was evaluated in 18 roots for each of five genotypes: Col-0 (wild-type), *exo84b-1*, *nerd1-1*, *nerd1-2*, and *nerd1-3* (Fig. 6; Supplementary Table S3). Root hairs in *nerd1* mutants, as in wild-type, grow out of the apical end of the trichoblasts at a single location, thus indicating that root hair initiation *per se* is unaffected in *nerd1* mutants. However, *nerd1* root hairs are often more bulbous, with wider bases and shanks on average, compared with Col-0 (Fig. 6E). Because *nerd1* roots exhibit both wild-type and bulbous root hair morphologies, high standard deviations are associated with *nerd1* root hair measurements. Rupture of root hairs, evidenced by the extrusion of cytoplasmic contents into the medium from the root hair tip, is also notable in *nerd1* mutants (Fig. 6C and D), occurring in 26–34% of root hairs evaluated, compared with a rare incidence (0–1.8%) in Col-0 or *exo84b-1* (Fig. 6F; Supplementary Table S3). Average root hair length for *nerd1* root hairs growing within the medium is similar to that in wild-type (*nerd1-1*: 227 μm; *nerd1-3*: 247 μm; WT: 230 μm), even though rupture presumably stopped growth in some of the mutant root hairs. Overall, *nerd1* root hair morphology is consistent with a role for NERD1 in establishing the structural stability, and perhaps limiting compliance, of the cell wall in growing root hairs. Increased compliance of the cell wall upon loss of NERD1 function might allow for more rapid expansion, leading to increased root hair lengths. But such an effect might also make root hairs vulnerable to bulbous expansion and bursting, as is observed.

## Discussion

Secretory events upon which plant growth and development depend are manifested and regulated by a complex network of cellular components interacting both directly and indirectly. Facilitating secretion in many circumstances is an octameric protein complex, the exocyst (Cole and Fowler, 2006; Žárský *et al.*, 2013; Kulich *et al.*, 2015; Vukašinović and Žárský, 2016). To search for unknown components of the exocyst-mediated secretory network, we used a second-site enhancer screen, and identified *nerd1* mutants via their genetic interaction with exocyst mutants, influencing primary root growth, root hair expansion, and hypocotyl elongation in Arabidopsis. Notably, the interaction between NERD1 and the exocyst appears to be indirect, and thus would not have been detected by other methods, e.g. yeast two-hybrid screening. Mutation of *NERD1* leads to a shortened root meristem and reduced cell elongation in both the primary root and etiolated hypocotyls. These defects are also seen in plants with mutations affecting exocyst components, but are synergistically accentuated when both *nerd1* and exocyst mutations are combined. Additionally, mutations of both *NERD1* and components of the exocyst affect root hair morphology. The nearly ubiquitous expression of NERD1 throughout the plant (similar to that of most exocyst components) and its conservation throughout the plant kingdom underline its potential importance in a broader context. Notably, we were unable to generate a doubly homozygous *nerd1*/exocyst mutant plant from the self-cross of a double heterozygote combining *nerd1-3* with severe exocyst mutants (i.e. *exo84b-1* and *sec8-3*; 0 out of 58 and 0 out of 66 individuals genotyped from *nerd*/exocyst segregating populations, respectively; *P*<0.05 by chi-square test for each). This suggests that mutations of *NERD1* combined with severe exocyst mutations lead to lethality due to very early developmental defects.

We initially hypothesized that NERD1 directly interacts with the exocyst at the plasma membrane, where exocyst components are known to localize (Fendrych *et al.*, 2010). However, the majority of proteomic studies identify NERD1 in the ER or Golgi (Parsons *et al.*, 2012; Nikolovski *et al.*, 2014; Heard *et al.*, 2015), and not in the plasma membrane (Mitra *et al.*, 2009). Direct examination of the functional NERD1–GFP fusion validated its prominent localization in the Golgi (Fig. 2; Supplementary Fig. S6), in contrast to the preferential association of exocyst components with the plasma membrane (Fig. 3), suggesting that NERD1–exocyst interaction is indirect. Although it remains possible that NERD1 is present at the plasma membrane transiently or at a low level, the most likely interpretation of our results is that the synergistic genetic interaction between NERD1 and exocyst components does not involve direct physical contact.

One alternative hypothesis that does not rely on direct contact to explain the observed NERD1–exocyst genetic interaction is that NERD1 is required for correct exocyst localization; or vice versa, NERD1 is a cargo for exocyst-mediated trafficking. A few specific cargos requiring the plant exocyst for correct delivery have been identified (pectinacious mucilage in Arabidopsis seed coats (Kulich *et al.*, 2010); callose in leaf trichomes (Kulich *et al.*, 2015); and the integral plasma membrane proteins PEN3/ABCG36 and NIP5;1 (Mao *et al.*, 2016)). However, no mislocalization of fluorescently tagged exocyst components in *nerd1* mutants, or of NERD1–GFP in exocyst mutants, was observed (Fig. 3), arguing against this possibility. On the other hand, these experiments do not exclude genetic interaction via a currently unknown cargo that requires both NERD1 and exocyst-mediated vesicle transport for its proper function.

The localization of NERD1 to the Golgi, the site of synthesis of non-cellulose polysaccharides incorporated into the cell wall matrix (e.g. pectin and hemicellulose; Driouich *et al.*, 2012; Kim and Brandizzi, 2016), is tantalizing. NERD1 could be involved in the formation or function of *trans*-Golgi-localized protein complexes, such as the ECHIDNA/YIP4 complex that plays a role in post-Golgi secretion of pectin and hemicellulose to the cell wall, and which also influences cell elongation in roots and hypocotyls (Gendre *et al.*, 2013). However, we currently favor a working hypothesis, based on predicted structure, in which NERD1 directly affects a cell wall matrix polysaccharide, glycoprotein or proteoglycan that is ultimately secreted via exocyst-mediated trafficking to influence cell wall growth and expansion. Most of NERD1 is predicted to be located in the Golgi lumen, folded into a β-propeller-like tertiary structure that could serve as a scaffold for interactions with polysaccharides. Homology modeling suggests that this lumenal portion of NERD1 resembles proteins that interact with polysaccharides, i.e. lectins, integrins, and carbohydrate binding proteins. Perhaps most notably, threading programs identify certain NERD1 regions as similar to bacterial RGI pectin lyases, and to a lesser extent xyloglucanases (Supplmentary Dataset S2). Interestingly, RGI pectin lyases are activated by calcium, consistent with the calcium-binding pocket predicted for NERD1. Examination of the cell wall in *nerd1* roots by, for example, histochemical staining for specific components should help test the hypothesis that NERD1’s impact arises from a role influencing cell wall structure.

It is noteworthy that the phenotypes observed in *nerd1* mutants are consistent with cell wall pectins as a target of NERD1 activity. Altering the synthesis of pectic polysaccharides is known to cause a dwarfed phenotype with developmental defects that include shorter primary roots and reduced elongation of etiolated hypocotyls (Reboul *et al.*, 2011), reminiscent of defects observed in *nerd1* mutants. RGI pectins impact the same aspects of root hair morphology (e.g. swelling and branching) as those altered in *nerd1* mutants (Diet *et al.*, 2006; Reboul *et al.*, 2011). The accelerated cell elongation phase observed in etiolated hypocotyls is the result of cell wall modification, and in particular has been associated with altered pectins (Derbyshire *et al.*, 2007; Pelletier *et al.*, 2010), possibly independent of cellulose synthesis. Thus, a role of NERD1 in the synthesis or modification of cell wall pectins might explain the range of phenotypes observed in *nerd1* mutants and deserves further investigation.

Intriguingly, the effect of NERD1 on cell expansion is not uniform throughout development: *nerd1* mutations result in reduced cell elongation in the root tip and etiolated hypocotyl, but increased elongation of root hairs, along with increased likelihood of rupture at the growing root hair tip. It is also of note that a mutation altering Arabidopsis EXO70C2, another potential indirect exocyst interactor, leads to a similar phenotype in the tip-growing pollen tube: more rapid growth and increased tube rupture (Synek *et al.*, 2017). The contrasting effects of *nerd1* mutants on root hair tip growth versus primary root cell elongation could manifest because the cell wall matrices in the two cell types are structurally distinct from each other, generated by fundamentally different processes: delivery of non-cellulosic cell wall components to a narrowly focused region versus more broadly distributed modification of a preexisting cell wall matrix, respectively. Additional indirect evidence that cell walls are differentially altered is the increased incidence of bulging in *nerd1* root hair shafts, although neither bulging nor rupture is characteristic of cells in mutant root meristematic and elongation zones. In the root, the composition and structure of the cell wall changes as cells progress through division, elongation, and differentiation zones. For example, the rhamnogalacturonan I pectin in cell walls is modified during the transition from cell proliferation to cell elongation in roots (Willats *et al.*, 1999), and pectins in root hairs are structurally distinct from those in the lateral cell walls elsewhere in the primary root (Muszyński *et al.*, 2015). Thus, the composition of cell wall components available for interaction with NERD1 within the Golgi likely varies as root cells progress from elongation to differentiation. Such differences could alter NERD1’s impact on cell wall extensibility, elongation, and fragility in a developmentally dependent manner. Revealing the molecular function of NERD1 should help define how it is integrated with secretory system to help determine developmentally stage-specific patterns of cell growth and expansion.

## Supplementary data

Supplementary data are available at *JXB* online.

Methods. *In silico* prediction of NERD1 tertiary structure, with references.

Dataset S1. Alignment of *NERD1* homologs.

Dataset S2. Threading analysis of NERD1.

Dataset S3. Alignment of NERD1 homologs, Jalview format.

Fig. S1. SHOREmap identification of *nerd1* candidate genes.

Fig. S2. Images of root hair morphology in *nerd1.*

Fig. S3. RT-PCR for *nerd1* mutants.

Fig. S4. Diagram of predicted domains in NERD1 protein.

Fig. S5. Model depicting predicted tertiary structure of NERD1.

Fig. S6. Localization of NERD1–GFP to Golgi using NAG::Turq.

Fig. S7. Localization of NERD1–GFP changes in response to BFA.

Fig. S8. NERD1 acts synergistically with SEC8 to affect hypocotyl elongation.

Table S1. List of primers used for PCR and RT-PCR.

Table S2. Domains within NERD1 protein.

Table S3. Morphology of *nerd1* root hairs.

Supplementary Figures and Protocol S1

Supplementary Dataset S1

Supplementary Dataset S2

Supplementary Dataset S3

## References

[CIT0001] Alonso JM, Stepanova AN, Leisse TJ, et al 2003 Genome-wide insertional mutagenesis of *Arabidopsis thaliana*. Science 301, 653–657. 12893945 10.1126/science.1086391

[CIT0002] Biasini M, Bienert S, Waterhouse A, et al 2014 SWISS-MODEL: modelling protein tertiary and quaternary structure using evolutionary information. Nucleic Acids Research 42, W252–W258. 24782522 10.1093/nar/gku340 PMC4086089

[CIT0003] Bloch D, Pleskot R, Pejchar P, Potocký M, Trpkošová P, Cwiklik L, Vukašinović N, Sternberg H, Yalovsky S, Žárský V 2016 Exocyst SEC3 and phosphoinositides define sites of exocytosis in pollen tube initiation and growth. Plant Physiology 172, 980–1002. 27516531 10.1104/pp.16.00690 PMC5047084

[CIT0004] Cole RA, Fowler JE 2006 Polarized growth: maintaining focus on the tip. Current Opinion in Plant Biology 9, 579–588. 17010659 10.1016/j.pbi.2006.09.014

[CIT0005] Cole RA, McInally SA, Fowler JE 2014 Developmentally distinct activities of the exocyst enable rapid cell elongation and determine meristem size during primary root growth in Arabidopsis. BMC Plant Biology 14, 386. 25551204 10.1186/s12870-014-0386-0 PMC4302519

[CIT0006] Cole RA, Synek L, Zarsky V, Fowler JE 2005 SEC8, a subunit of the putative Arabidopsis exocyst complex, facilitates pollen germination and competitive pollen tube growth. Plant Physiology 138, 2005–2018. 16040664 10.1104/pp.105.062273 PMC1183391

[CIT0007] Cvrčková F, Grunt M, Bezvoda R, Hála M, Kulich I, Rawat A, Žárský V 2012 Evolution of the land plant exocyst complexes. Frontiers in Plant Science 3, 159. 22826714 10.3389/fpls.2012.00159 PMC3399122

[CIT0008] Derbyshire P, McCann MC, Roberts K 2007 Restricted cell elongation in Arabidopsis hypocotyls is associated with a reduced average pectin esterification level. BMC Plant Biology 7, 31. 17572910 10.1186/1471-2229-7-31 PMC1913053

[CIT0009] Diet A, Link B, Seifert GJ, Schellenberg B, Wagner U, Pauly M, Reiter WD, Ringli C 2006 The *Arabidopsis* root hair cell wall formation mutant *lrx1* is suppressed by mutations in the *RHM1* gene encoding a UDP-L-rhamnose synthase. The Plant Cell 18, 1630–1641. 16766693 10.1105/tpc.105.038653 PMC1488927

[CIT0010] Drakakaki G, Dandekar A 2013 Protein secretion: how many secretory routes does a plant cell have?Plant Science 203–204, 74–78. 10.1016/j.plantsci.2012.12.017 23415330

[CIT0011] Driouich A, Follet-Gueye ML, Bernard S, Kousar S, Chevalier L, Vicré-Gibouin M, Lerouxel O 2012 Golgi-mediated synthesis and secretion of matrix polysaccharides of the primary cell wall of higher plants. Frontiers in Plant Science 3, 79. 22639665 10.3389/fpls.2012.00079 PMC3355623

[CIT0012] Ebine K, Ueda T 2015 Roles of membrane trafficking in plant cell wall dynamics. Frontiers in Plant Science 6, 878. 26539200 10.3389/fpls.2015.00878 PMC4609830

[CIT0013] Emanuelsson O, Nielsen H, Brunak S, von Heijne G 2000 Predicting subcellular localization of proteins based on their N-terminal amino acid sequence. Journal of Molecular Biology 300, 1005–1016. 10891285 10.1006/jmbi.2000.3903

[CIT0014] Fendrych M, Synek L, Pecenková T, Drdová EJ, Sekeres J, de Rycke R, Nowack MK, Zársky V 2013 Visualization of the exocyst complex dynamics at the plasma membrane of *Arabidopsis thaliana*. Molecular Biology of the Cell 24, 510–520. 23283982 10.1091/mbc.E12-06-0492 PMC3571873

[CIT0015] Fendrych M, Synek L, Pecenková T, et al 2010 The Arabidopsis exocyst complex is involved in cytokinesis and cell plate maturation. The Plant Cell 22, 3053–3065. 20870962 10.1105/tpc.110.074351 PMC2965533

[CIT0016] Gendre D, McFarlane HE, Johnson E, Mouille G, Sjödin A, Oh J, Levesque-Tremblay G, Watanabe Y, Samuels L, Bhalerao RP 2013 *Trans*-Golgi network localized ECHIDNA/Ypt interacting protein complex is required for the secretion of cell wall polysaccharides in *Arabidopsis*. The Plant Cell 25, 2633–2646. 23832588 10.1105/tpc.113.112482 PMC3753388

[CIT0017] Grennan AK 2006 Genevestigator. Facilitating web-based gene-expression analysis. Plant Physiology 141, 1164–1166. 16896229 10.1104/pp.104.900198 PMC1533917

[CIT0018] Hála M, Cole R, Synek L, et al 2008 An exocyst complex functions in plant cell growth in Arabidopsis and tobacco. The Plant Cell 20, 1330–1345. 18492870 10.1105/tpc.108.059105 PMC2438459

[CIT0019] Heard W, Sklenář J, Tomé DF, Robatzek S, Jones AM 2015 Identification of regulatory and cargo proteins of endosomal and secretory pathways in *Arabidopsis thaliana* by proteomic dissection. Molecular & Cellular Proteomics 14, 1796–1813. 25900983 10.1074/mcp.M115.050286 PMC4587325

[CIT0020] Hong D, Jeon BW, Kim SY, Hwang JU, Lee Y 2016 The ROP2-RIC7 pathway negatively regulates light-induced stomatal opening by inhibiting exocyst subunit Exo70B1 in Arabidopsis. New Phytologist 209, 624–635. 26451971 10.1111/nph.13625

[CIT0021] Jin Y, Sultana A, Gandhi P, Franklin E, Hamamoto S, Khan AR, Munson M, Schekman R, Weisman LS 2011 Myosin V transports secretory vesicles via a Rab GTPase cascade and interaction with the exocyst complex. Developmental Cell 21, 1156–1170. 22172676 10.1016/j.devcel.2011.10.009 PMC3241923

[CIT0022] Kalmbach L, Hématy K, De Bellis D, Barberon M, Fujita S, Ursache R, Daraspe J, Geldner N 2017 Transient cell-specific EXO70A1 activity in the CASP domain and Casparian strip localization. Nature Plants 3, 17058. 28436943 10.1038/nplants.2017.58

[CIT0023] Källberg M, Wang H, Wang S, Peng J, Wang Z, Lu H, Xu J 2012 Template-based protein structure modeling using the RaptorX web server. Nature Protocols 7, 1511–1522. 22814390 10.1038/nprot.2012.085 PMC4730388

[CIT0024] Kelley LA, Mezulis S, Yates CM, Wass MN, Sternberg MJ 2015 The Phyre2 web portal for protein modeling, prediction and analysis. Nature Protocols 10, 845–858. 25950237 10.1038/nprot.2015.053 PMC5298202

[CIT0025] Kersey PJ, Allen JE, Armean I, et al 2016 Ensembl genomes 2016: more genomes, more complexity. Nucleic Acids Research 44, D574–D580. 26578574 10.1093/nar/gkv1209 PMC4702859

[CIT0026] Kim SJ, Brandizzi F 2016 The plant secretory pathway for the trafficking of cell wall polysaccharides and glycoproteins. Glycobiology 26, 940–949. 27072815 10.1093/glycob/cww044

[CIT0027] Kopec KO, Lupas AN 2013 β-Propeller blades as ancestral peptides in protein evolution. PLoS ONE 8, e77074. 24143202 10.1371/journal.pone.0077074 PMC3797127

[CIT0028] Kulich I, Cole R, Drdová E, Cvrcková F, Soukup A, Fowler J, Žárský V 2010 Arabidopsis exocyst subunits SEC8 and EXO70A1 and exocyst interactor ROH1 are involved in the localized deposition of seed coat pectin. New Phytologist 188, 615–625. 20618910 10.1111/j.1469-8137.2010.03372.x

[CIT0029] Kulich I, Pečenková T, Sekereš J, Smetana O, Fendrych M, Foissner I, Höftberger M, Žárský V 2013 Arabidopsis exocyst subcomplex containing subunit EXO70B1 is involved in autophagy-related transport to the vacuole. Traffic 14, 1155–1165. 23944713 10.1111/tra.12101

[CIT0030] Kulich I, Vojtíková Z, Glanc M, Ortmannová J, Rasmann S, Žárský V 2015 Cell wall maturation of Arabidopsis trichomes is dependent on exocyst subunit EXO70H4 and involves callose deposition. Plant Physiology 168, 120–131. 25767057 10.1104/pp.15.00112 PMC4424025

[CIT0031] Lavy M, Bloch D, Hazak O, Gutman I, Poraty L, Sorek N, Sternberg H, Yalovsky S 2007 A novel ROP/RAC effector links cell polarity, root-meristem maintenance, and vesicle trafficking. Current Biology 17, 947–952. 17493810 10.1016/j.cub.2007.04.038

[CIT0032] Li S, Chen M, Yu D, Ren S, Sun S, Liu L, Ketelaar T, Emons AM, Liu CM 2013 EXO70A1-mediated vesicle trafficking is critical for tracheary element development in Arabidopsis. The Plant Cell 25, 1774–1786. 23709627 10.1105/tpc.113.112144 PMC3694705

[CIT0033] Li S, van Os GM, Ren S, Yu D, Ketelaar T, Emons AM, Liu CM 2010 Expression and functional analyses of EXO70 genes in Arabidopsis implicate their roles in regulating cell type-specific exocytosis. Plant Physiology 154, 1819–1830. 20943851 10.1104/pp.110.164178 PMC2996038

[CIT0034] Liu D, Novick P 2014 Bem1p contributes to secretory pathway polarization through a direct interaction with Exo70p. The Journal of Cell Biology 207, 59–72. 25313406 10.1083/jcb.201404122 PMC4195821

[CIT0035] Liu J, Zhao Y, Sun Y, He B, Yang C, Svitkina T, Goldman YE, Guo W 2012 Exo70 stimulates the Arp2/3 complex for lamellipodia formation and directional cell migration. Current Biology 22, 1510–1515. 22748316 10.1016/j.cub.2012.05.055 PMC3427469

[CIT0036] Luschnig C, Vert G 2014 The dynamics of plant plasma membrane proteins: PINs and beyond. Development 141, 2924–2938. 25053426 10.1242/dev.103424

[CIT0037] Mani R, Onge RPS, Hartman JL, Giaever G, Roth FP 2008 Defining genetic interaction. Proceedings of the National Academy of Sciences, USA 105, 3461–3466. 10.1073/pnas.0712255105 PMC2265146 18305163

[CIT0038] Mao H, Nakamura M, Viotti C, Grebe M 2016 A framework for lateral membrane trafficking and polar tethering of the PEN3 ATP-binding cassette transporter. Plant Physiology 172, 2245–2260. 27803190 10.1104/pp.16.01252 PMC5129716

[CIT0039] Mitra SK, Walters BT, Clouse SD, Goshe MB 2009 An efficient organic solvent based extraction method for the proteomic analysis of Arabidopsis plasma membranes. Journal of Proteome Research 8, 2752–2767. 19334764 10.1021/pr801044y

[CIT0040] Mukherjee D, Sen A, Aguilar RC 2014 RhoGTPase-binding proteins, the exocyst complex and polarized vesicle trafficking. Small GTPases 5, e28453. 24691289 10.4161/sgtp.28453 PMC4114650

[CIT0041] Muszyński A, O’Neill MA, Ramasamy E, et al 2015 Xyloglucan, galactomannan, glucuronoxylan, and rhamnogalacturonan I do not have identical structures in soybean root and root hair cell walls. Planta 242, 1123–1138. 26067758 10.1007/s00425-015-2344-y

[CIT0042] Nikolovski N, Shliaha PV, Gatto L, Dupree P, Lilley KS 2014 Label-free protein quantification for plant Golgi protein localization and abundance. Plant Physiology 166, 1033–1043. 25122472 10.1104/pp.114.245589 PMC4213074

[CIT0043] Oda Y, Iida Y, Nagashima Y, Sugiyama Y, Fukuda H 2015 Novel coiled-coil proteins regulate exocyst association with cortical microtubules in xylem cells via the conserved oligomeric golgi-complex 2 protein. Plant & Cell Physiology 56, 277–286. 25541219 10.1093/pcp/pcu197

[CIT0044] Park E, Nebenführ A 2013 Myosin XIK of *Arabidopsis thaliana* accumulates at the root hair tip and is required for fast root hair growth. PLoS ONE 8, e76745. 24116145 10.1371/journal.pone.0076745 PMC3792037

[CIT0045] Parsons HT, Christiansen K, Knierim B, et al 2012 Isolation and proteomic characterization of the Arabidopsis Golgi defines functional and novel components involved in plant cell wall biosynthesis. Plant Physiology 159, 12–26. 22430844 10.1104/pp.111.193151 PMC3375956

[CIT0046] Pelletier S, Van Orden J, Wolf S, et al 2010 A role for pectin de-methylesterification in a developmentally regulated growth acceleration in dark-grown Arabidopsis hypocotyls. New Phytologist 188, 726–739. 20819179 10.1111/j.1469-8137.2010.03409.x

[CIT0047] Peremyslov VV, Cole RA, Fowler JE, Dolja VV 2015 Myosin-powered membrane compartment drives cytoplasmic streaming, cell expansion and plant development. PLoS ONE 10, e0139331. 26426395 10.1371/journal.pone.0139331 PMC4591342

[CIT0048] Peremyslov VV, Klocko AL, Fowler JE, Dolja VV 2012 Arabidopsis myosin XI-K localizes to the motile endomembrane vesicles associated with F-actin. Frontiers in Plant Science 3, 184. 22969781 10.3389/fpls.2012.00184 PMC3432474

[CIT0049] Peremyslov VV, Prokhnevsky AI, Avisar D, Dolja VV 2008 Two class XI myosins function in organelle trafficking and root hair development in Arabidopsis. Plant Physiology 146, 1109–1116. 18178669 10.1104/pp.107.113654 PMC2259062

[CIT0050] Pleskot R, Cwiklik L, Jungwirth P, Žárský V, Potocký M 2015 Membrane targeting of the yeast exocyst complex. Biochimica et Biophysica Acta 1848, 1481–1489. 25838123 10.1016/j.bbamem.2015.03.026

[CIT0051] Poulsen CP, Dilokpimol A, Mouille G, Burow M, Geshi N 2014 Arabinogalactan glycosyltransferases target to a unique subcellular compartment that may function in unconventional secretion in plants. Traffic 15, 1219–1234. 25074762 10.1111/tra.12203 PMC4285201

[CIT0052] Reboul R, Geserick C, Pabst M, Frey B, Wittmann D, Lütz-Meindl U, Léonard R, Tenhaken R 2011 Down-regulation of UDP-glucuronic acid biosynthesis leads to swollen plant cell walls and severe developmental defects associated with changes in pectic polysaccharides. The Journal of Biological Chemistry 286, 39982–39992. 21949134 10.1074/jbc.M111.255695 PMC3220558

[CIT0053] Ritzenthaler C, Nebenführ A, Movafeghi A, Stussi-Garaud C, Behnia L, Pimpl P, Staehelin LA, Robinson DG 2002 Reevaluation of the effects of brefeldin A on plant cells using tobacco Bright Yellow 2 cells expressing Golgi-targeted green fluorescent protein and COPI antisera. The Plant Cell 14, 237–261. 11826310 10.1105/tpc.010237 PMC150562

[CIT0054] Robinson DG, Ding Y, Jiang L 2016 Unconventional protein secretion in plants: a critical assessment. Protoplasma 253, 31–43. 26410830 10.1007/s00709-015-0887-1

[CIT0055] Rosso MG, Li Y, Strizhov N, Reiss B, Dekker K, Weisshaar B 2003 An *Arabidopsis thaliana* T-DNA mutagenized population (GABI-Kat) for flanking sequence tag-based reverse genetics. Plant Molecular Biology 53, 247–259. 14756321 10.1023/B:PLAN.0000009297.37235.4a

[CIT0056] Rybak K, Steiner A, Synek L, et al 2014 Plant cytokinesis is orchestrated by the sequential action of the TRAPPII and exocyst tethering complexes. Developmental Cell 29, 607–620. 24882377 10.1016/j.devcel.2014.04.029

[CIT0057] Schneeberger K, Ossowski S, Lanz C, Juul T, Petersen AH, Nielsen KL, Jørgensen JE, Weigel D, Andersen SU 2009 SHOREmap: simultaneous mapping and mutation identification by deep sequencing. Nature Methods 6, 550–551. 19644454 10.1038/nmeth0809-550

[CIT0058] Sekereš J, Pejchar P, Šantrůček J, Vukašinović N, Žárský V, Potocký M 2017 Analysis of exocyst subunit EXO70 family reveals distinct membrane polar domains in tobacco pollen tubes. Plant Physiology 173, 1659–1675. 28082718 10.1104/pp.16.01709 PMC5338673

[CIT0059] Synek L, Schlager N, Eliás M, Quentin M, Hauser MT, Žárský V 2006 AtEXO70A1, a member of a family of putative exocyst subunits specifically expanded in land plants, is important for polar growth and plant development. The Plant Journal 48, 54–72. 16942608 10.1111/j.1365-313X.2006.02854.x PMC2865999

[CIT0060] Synek L, Vukašinović N, Kulich I, Hála M, Aldorfová K, Fendrych M, Žárský V 2017 EXO70C2 is a key regulatory factor for optimal tip growth of pollen. Plant Physiology 174, 223–240. 28356503 10.1104/pp.16.01282 PMC5411130

[CIT0061] Thapa N, Sun Y, Schramp M, Choi S, Ling K, Anderson RA 2012 Phosphoinositide signaling regulates the exocyst complex and polarized integrin trafficking in directionally migrating cells. Developmental Cell 22, 116–130. 22264730 10.1016/j.devcel.2011.10.030 PMC3266520

[CIT0062] van de Meene AML, Doblin MS, Bacic A 2017 The plant secretory pathway seen through the lens of the cell wall. Protoplasma 254, 75–94. 26993347 10.1007/s00709-016-0952-4

[CIT0063] Vukašinović N, Žárský V 2016 Tethering complexes in the arabidopsis endomembrane system. Frontiers in Cell and Developmental Biology 4, 46. 27243010 10.3389/fcell.2016.00046 PMC4871884

[CIT0064] Wass MN, Kelley LA, Sternberg MJ 2010 3DLigandSite: predicting ligand-binding sites using similar structures. Nucleic Acids Research 38, W469–W473. 20513649 10.1093/nar/gkq406 PMC2896164

[CIT0065] Wen TJ, Hochholdinger F, Sauer M, Bruce W, Schnable PS 2005 The *roothairless1* gene of maize encodes a homolog of *sec3*, which is involved in polar exocytosis. Plant Physiology 138, 1637–1643. 15980192 10.1104/pp.105.062174 PMC1176433

[CIT0066] Willats WG, Steele-King CG, Marcus SE, Knox JP 1999 Side chains of pectic polysaccharides are regulated in relation to cell proliferation and cell differentiation. The Plant Journal 20, 619–628. 10652134 10.1046/j.1365-313x.1999.00629.x

[CIT0067] Wu B, Guo W 2015 The exocyst at a glance. Journal of Cell Science 128, 2957–2964. 26240175 10.1242/jcs.156398 PMC4541039

[CIT0068] Žárský V, Kulich I, Fendrych M, Pečenková T 2013 Exocyst complexes multiple functions in plant cells secretory pathways. Current Opinion in Plant Biology 16, 726–733. 24246229 10.1016/j.pbi.2013.10.013

[CIT0069] Zhang C, Brown MQ, van de Ven W, et al 2016 Endosidin2 targets conserved exocyst complex subunit EXO70 to inhibit exocytosis. Proceedings of the National Academy of Sciences, USA 113, E41–50. 10.1073/pnas.1521248112 PMC4711834 26607451

[CIT0070] Zhang X, Pumplin N, Ivanov S, Harrison MJ 2015 EXO70I is required for development of a sub-domain of the periarbuscular membrane during arbuscular mycorrhizal symbiosis. Current Biology 25, 2189–2195. 26234213 10.1016/j.cub.2015.06.075

[CIT0071] Zhang ZJ, Peck SC 2011 Simplified enrichment of plasma membrane proteins for proteomic analyses in *Arabidopsis thaliana*. Proteomics 11, 1780–1788. 21433285 10.1002/pmic.201000648

