# Supplementary material for: A broadly conserved NERD genetically interacts with the exocyst to affect root growth and cell expansion
Source: J Exp Bot. 2018 May 2;69(15):3625–37. doi: 10.1093/jxb/ery162 (PMC6022600; doi:10.1093/jxb/ery162)
Supplement: Supplementary Figures and Protocol S1 [file ery162_suppl_supplementary_figures_protocol_s1.pdf]

A broadly-conserved NERD interacts with the exocyst to affect root growth and cell expansion

SUPPLEMENTARY INFORMATION:

Supplementary Figures S1-S8

Supplementary Tables S1-S3

Supplementary Methods and References

Rex Cole, Valera Peremyslov, Savanah Van Why, Ibrahim Moussaoui, Ann Ketter, Renee Cool, Matthew Andres Moreno, Zuzana Vejlupkova, Valerian Dolja, and John E. Fowler

Department of Botany and Plant Pathology and Center for Genome Research and Biocomputing, Oregon State University, Corvallis, OR 97331, USA

Author for correspondence: John E. Fowler, Dept. of Botany & Plant Pathology, 2082 Cordley Hall, Oregon State University, Corvallis, OR 97331; Phone: 541 737 5307; FAX: 541 737 3573; email: fowlerj@science.oregonstate.edu

Running title: NERD1 interacts with the exocyst in root development

Col-0

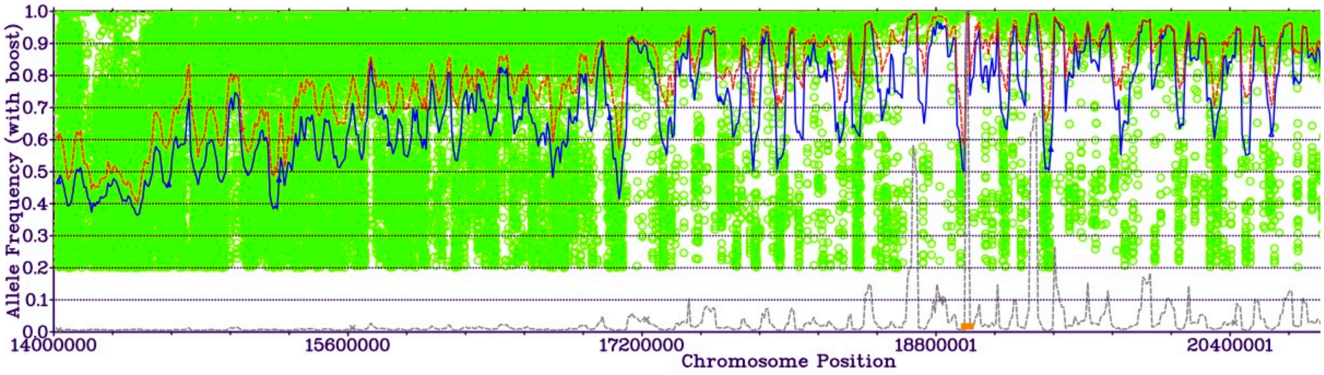

Ler-0

<u>Figure S1. SHOREmap identification of *NERD1* candidate genes</u>. The *nerd1-1* lesion maps between bp 18,850,000 – 19,050,000 of Chromosome 3. SHOREmap software assessed the frequency of *Col-0* and *Ler* SNPs in 150 pooled *nerd1-1* homozygous plants of an F2 population in a sliding window (red line; individual SNP frequencies are green circles). The orange bar at the bottom defines a region of 100% non-*Ler* SNPs, and therefore tightly linked to *nerd1-1*; non *Col-0* SNPs in this region are likely EMS lesions. ~65 protein-coding genes in this region were assessed for nucleotide differences (i.e., EMS lesions) from the progenitor *Col-0* sequence, identifying only two (At3g51050 and At3g52250).

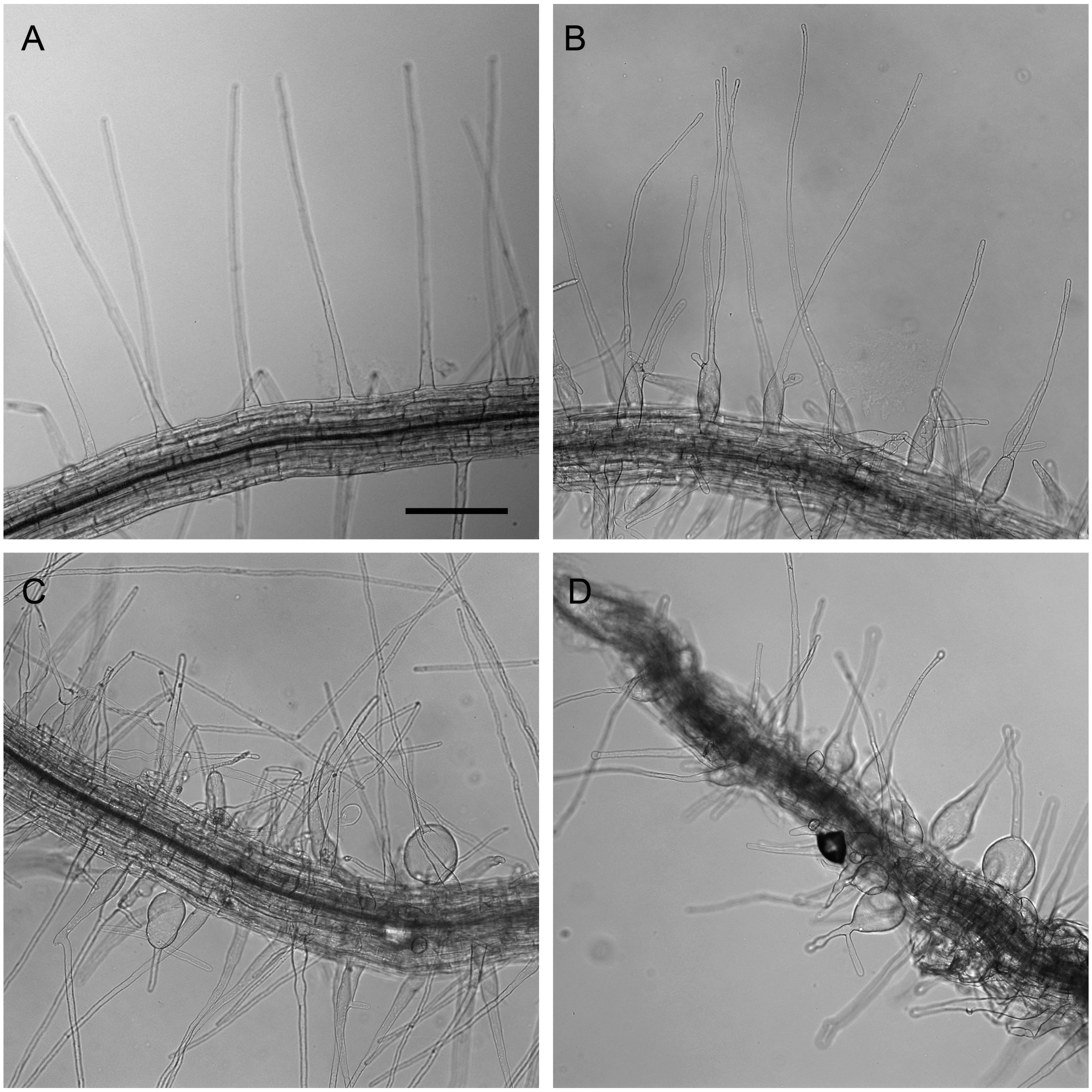

<u>Figure S2. Root hair morphology is altered in *nerd1* mutants.</u> Roots were grown on the surface of media on closed vertical plates for 5-7days. Root hairs were then examined microscopically on (A) Wild-type (Col-0), (B) *nerd1-1*, (C) *nerd1-2* roots, and (D) *nerd1-1 sec8-6* roots. The root hairs in the *nerd1* mutants show a variety of shapes, with representatives of the more abnormal shapes shown in B-D. Wild-type appearing root hairs are evident in seedlings with the *nerd1* mutation, including the *nerd1-1 sec8-6* double mutants, but *nerd1* is associated with increased bulging in some root hairs, primarily at the root hair base. Root hairs appear to more densely populate the surface of the root of the mutants, primarily because the root cells are shorter, and not because there are multiple root hairs per trichoblast cell. Root hair bursting is not clearly evident for roots growing on the surface of the media. See Figure 6 and Table S2 for quantitative assessment of altered morphology of root hairs grown within media. Size marker = 200 microns.

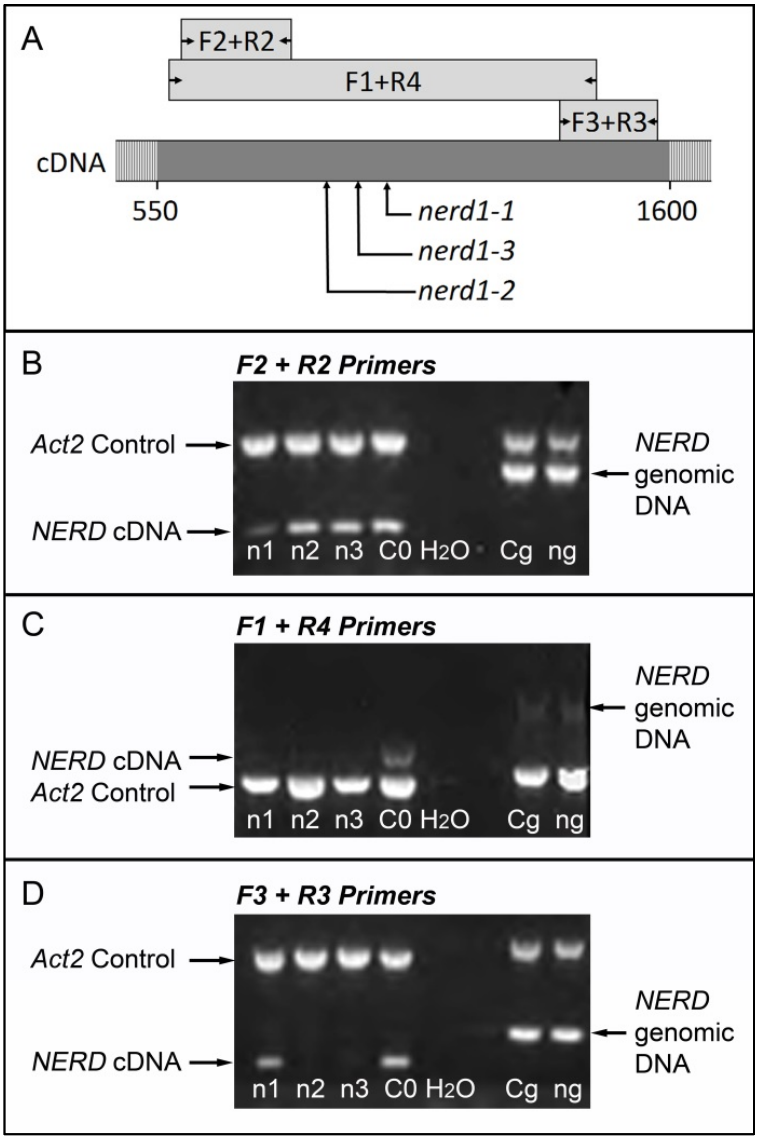

<u>Figure S3. RT-PCR results show altered transcripts in *nerd1* mutants.</u> cDNA was generated from root samples of *nerd1* and *Col-0* wild type roots. A. Diagram of portion of NERD1 cDNA showing location of *nerd1-1* point mutation, *nerd1-2* and *nerd1-3* T-DNA insertion mutations, and segments amplified by RT-PCR primers (F2+R2; F1+R4; F3+R3). B-D. Gel images of RT-PCR products from cDNA (n1=*nerd1-1,* n2=*nerd1-2*, n3=*nerd1-3*, C0=Col-0) and genomic DNA (Cg=Col-0, ng=*nerd1-1*). *ACTIN2* (At3g18780) served as an internal control. B. PCR amplification using primers 5' to the mutations (F2+R2) produced a product of the same size as Col-0 in the *nerd1* mutants, and different in size from genomic DNA. C. PCR amplification using primers spanning the region of the mutations (F1+R4) produced a product of expected size for Col-0 cDNA, but failed to produce a product in the *nerd* mutants. D. PCR amplification using primers 3' to the mutations (F3+R3) failed to produce a product with *nerd1-2* and *nerd1-3* cDNA templates. Primers F3+R3 did produce a product of the same size as Col-0 in the *nerd1-1* mutant, indicating that this portion of the transcript remained intact after this point mutation.

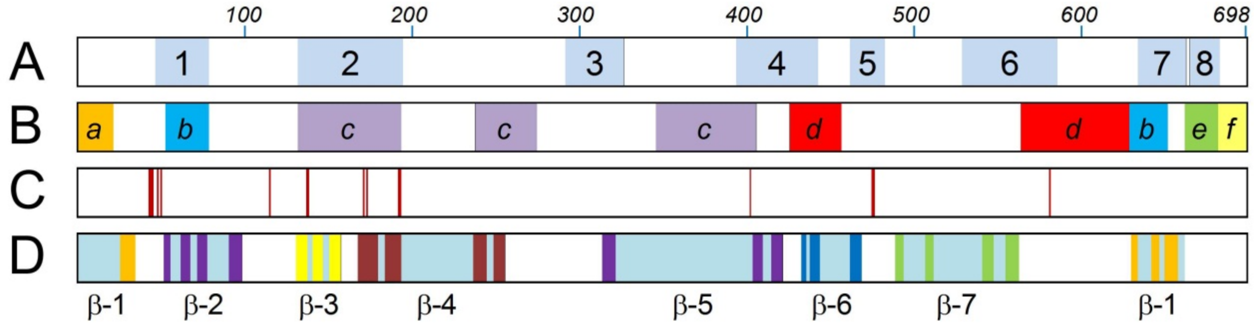

Figure S4. Domains identified in NERD1 by structural modeling or evolutionary conservation. A. Regions of the 698 amino acid protein that are particularly well-conserved throughout Angiosperms (See Data S1). B. Position of the domains listed in Table S1: a=signal peptide, b=FG-GAP domain, c=alcohol dehydrogenase-like domain, d=integrin-alpha like domain, e=transmembrane domain, and f=cytoplasmic domain. C. Location of residues predicted by RaptorX (Källberg *et al.*, 2012) to form a calcium binding pocket in a model of the folded protein. D. Position of the beta strands (in color) that form the seven blades (beta-sheets) of a predicted beta-propeller structure (see Figure S5). The light blue between the colored beta strands form loops that allow the beta strands to fold into the beta sheets of the propeller blades.

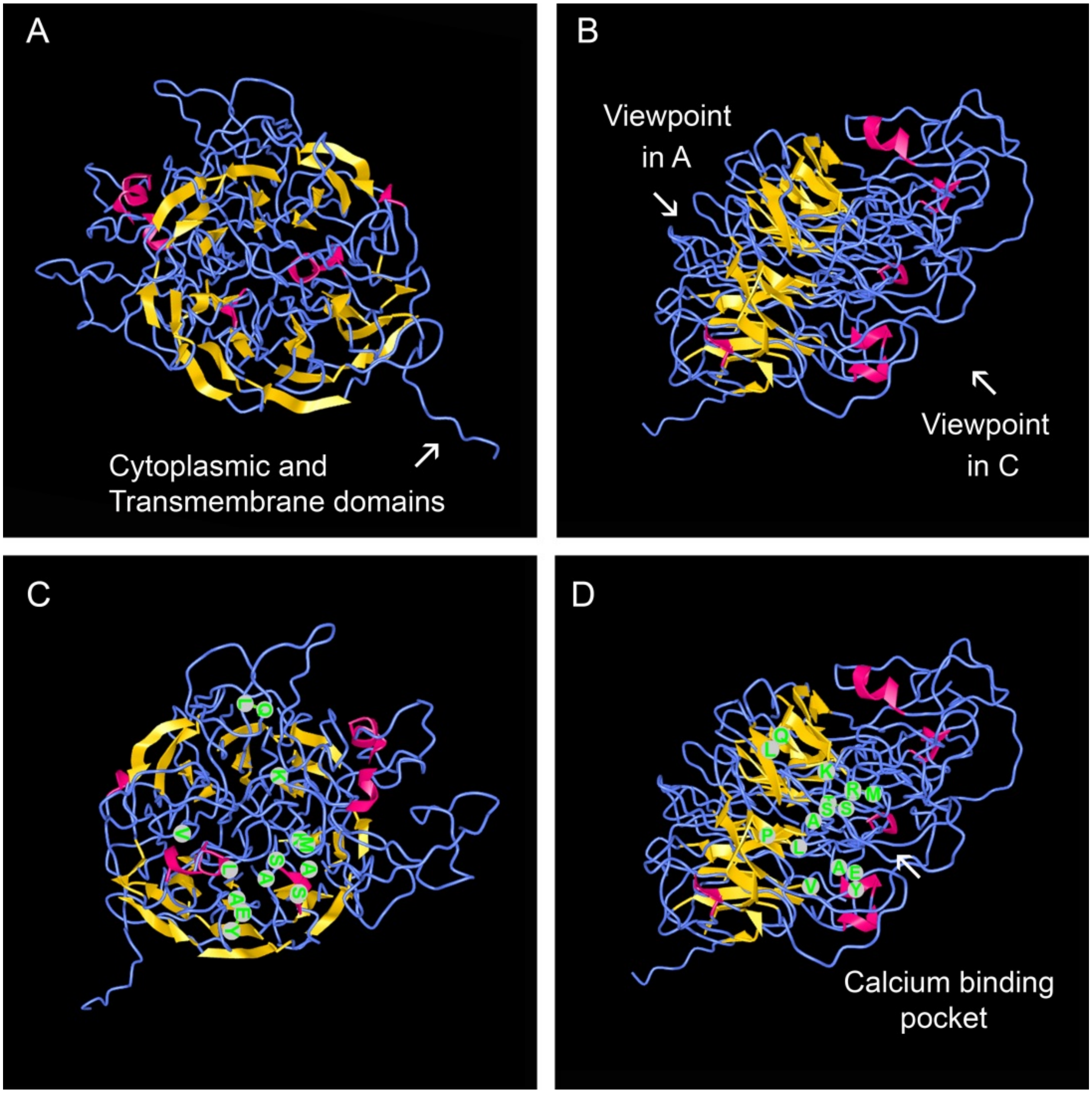

Figure S5. Model of predicted tertiary structure of NERD1. This model (A-D) is based upon RaptorX (Källberg *et al.*, 2012) analysis, utilizing comparison to five templates. (A) Beta strands shown in yellow are predicted to form a seven-bladed beta-propeller. Also visualized is the C-terminal tail that forms the transmembrane and cytosolic portion of the protein. (B) “Sideview” of the model showing a few short coils (pink) and loops (blue) extending from either face of the beta-propeller. (C) The side of the protein opposite to that shown in A with residues predicted to form a calcium binding pocket highlighted in green. (D) Side-view showing another perspective of the calcium binding pocket. Phyre2 (Kelley *et al.,* 2015) analysis predicted a similar model (see Data S2).

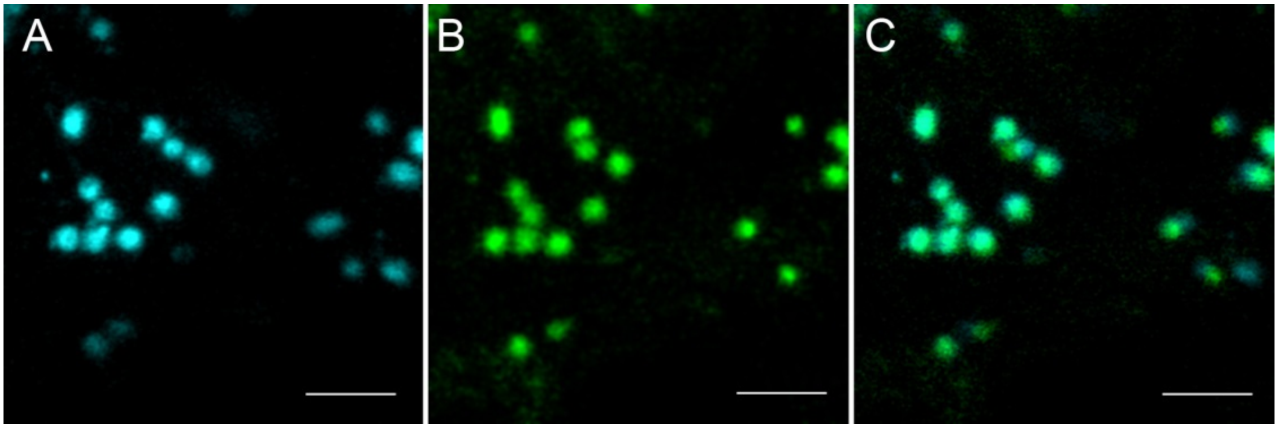

<u>Figure S6. Localization of NERD1-GFP to the Golgi</u>. NERD1-GFP co-localizes with the Golgi maker N-acetylglucosaminyl transferase (NAG)-Turqoise in leaf cells of *Nicotiana benthamiana*. (a) NAG-Turq; (b) NERD1-GFP; (c) merged image.
Bar = 5 mm.

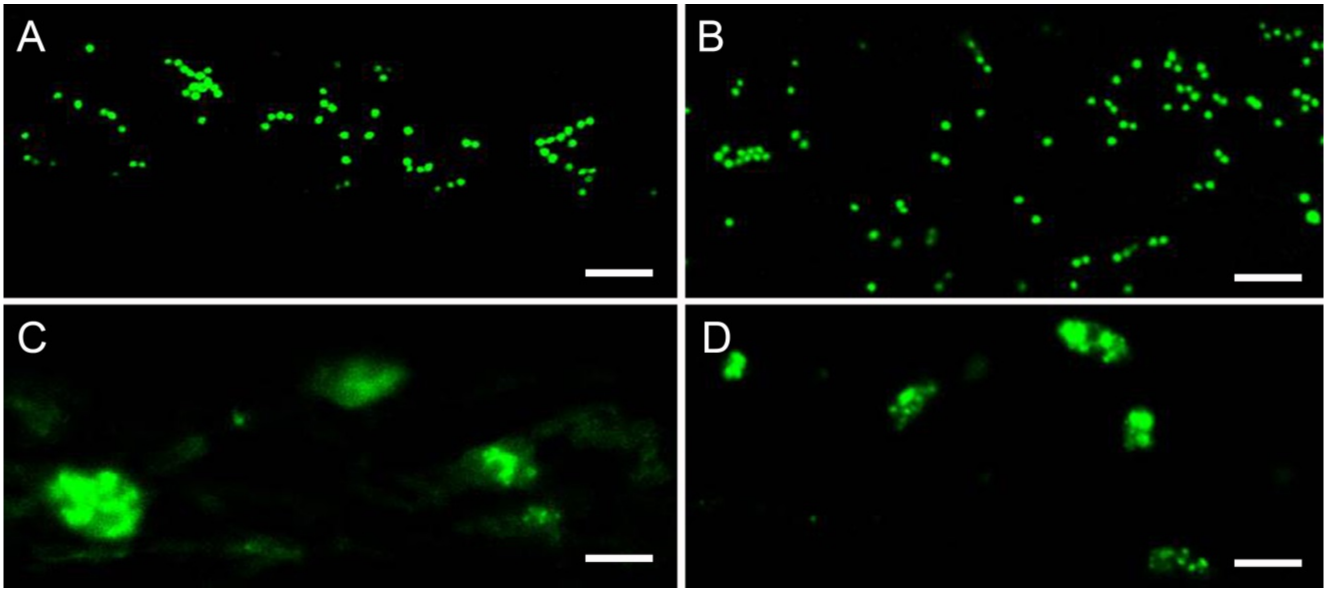

Figure S7. Localization of NERD1-GFP changes in response to BFA. Images of Arabidopsis root epidermal cells containing the positive control Golgi marker NAG-GFP (A and C) or NERD1-GFP (B and D). A and B show the markers as localized in untreated roots. C and D show that both markers coalesced into large fluorescent compartments after treatment with 50 μM BFA for 1.5 h.

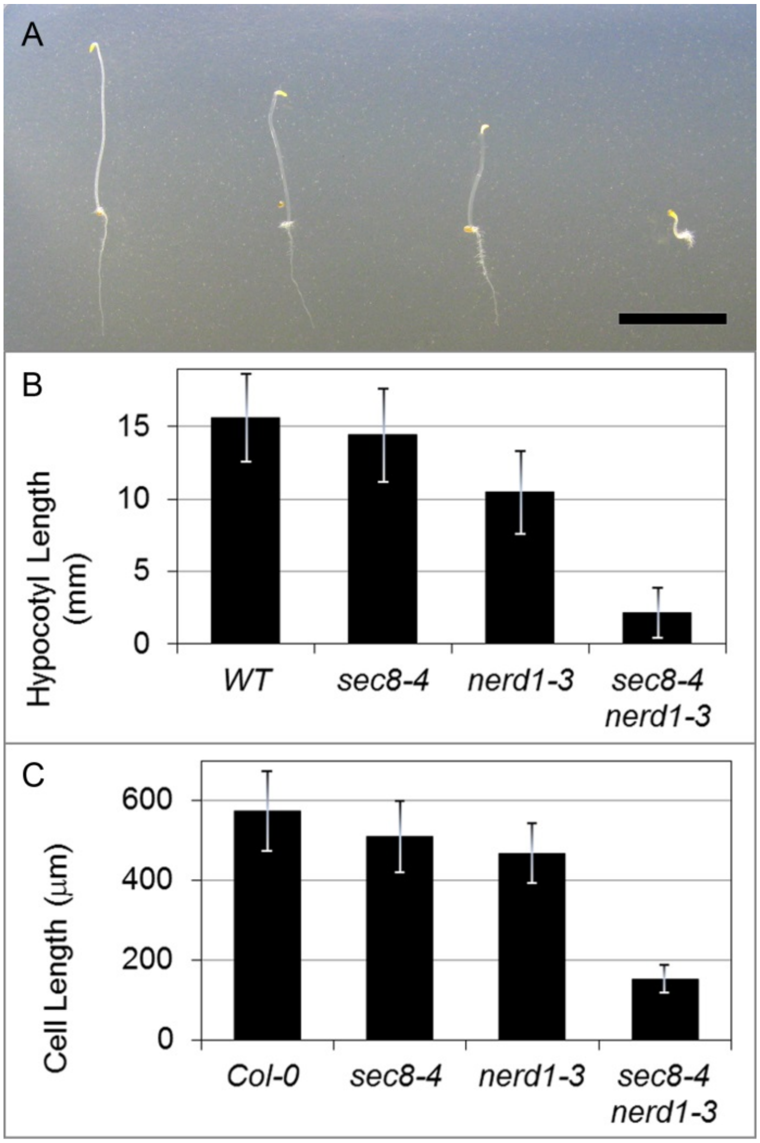

Figure S8. NERD1 acts synergistically with exocyst component SEC8 to affect hypocotyl elongation. A. Five day old etiolated seedlings, from left to right: wild type, *sec8-4*, *nerd1-3*, and *sec8-4 nerd1-3* double mutant (size marker = 1 cm). B. Hypocotyl lengths in five day old etiolated seedlings: wild type (n=196 hypocotyls), *sec8-4* (n=63), *nerd1-3* (n=54), and *sec8-4 nerd1-3* (n=54). C. Epidermal cell lengths in etiolated hypocotyls**:** Col-0 (n=46 cells), *sec8-4* (n=50), *nerd1-3* (n=99), and *sec8-4 nerd1-3* (n=106). [error bars = standard deviation]

Table S1. Primers used for PCR and RT-PCR

	PCR target	Primer Name	Primer sequence
Primers used for PCR genotyping	*nerd1-1*	N1a_F2	TTGAGTGCAGGGAATTTAGA
		N1a_R2	GCAAAGTGTTCGACCAGTAG
	nerd1-2 & *nerd1-3*	N1_F2	TTGTTGGTGGAATGACAGGA
		N1_R2	TGCATACCCTGCATCAACAT
	sec8-3	S8_F6	TGACTCGCCACACGAGGAACT
		S8_R6	TGGAGCCAGTCTTAAATGCACC
	sec8-4	S8_F5	CACGTAGGGAGGAGGGAATGG
		S8_R5	CCTGCTTCTCCTTTATGATTTCACC
	sec8-6	S8_F3	CACGTAGGGAGGAGGGAATGG
		S8_R3	TGGCAAACCAAAAGCCAAAAG
	exo70A1-2	X70A1e_L	CTAGACGTTTGCAGCATCCTA
		X70A1e_R	ATATGTGTAATGCATTGGAGAAGC
	exo84B-1	84b_Gabi_LP	TTGAAGGTTGACAAGCAGCC
		84b_Gabi_RP	TTGTTCTTGTGATCTTCTGGAG
	myo XI-K	SALK_067972-L	GGATCTCCCAACTTGAACTTTTC
		SALK_067972-R	GCAAGAGCAACTCAATTCTGG
	SALK T-DNA left border	LBb1	GCGTGGACCGCTTGCTGCAACT
		LBb1.3	ATTTTGCCGATTTCGGAAC
	GABI T-DNA left border	GABI_LB	GGGCTACACTGAATTGGTAGCTC
	NERD1-GFP	GFP-R	TCGCCGTCCAGCTCGACCAGG
		NERD1-F6	CTCTACTCTGGAGATCGCAAC
	in genomic *NERD1* and complementation construct	NtF1	AGGCCTCTGAAGATTCTGGA
		NtF2	TATGCATTTGCTGGCAAGAC
	In complementation construct only	NtR1	AAGTCGTGCTGCTTCATGTG
		NtR2	GAACTTCAGGGTCAGCTTGC
	3' of genomic *NERD1* only	NtR4	GAGAGGAAGAAAACTCTACGAG
Primer Pairs for RT-PCR of nerd1 mutants	*nerd1*: 5' to mutation	F2	CCACATAATGCACACCATAG
		R2	CAGCAAATGCATAGACAGAA
	nerd1: 3’ to mutation	F3	GAGATGGTGTTCTCGATCAT
		R3	TCCTCCATAGTGCAAGAAGT
	Across mutations	F1	AGGAGGATTTCCCACATAAT
		R4	CCGCTCACTACAGTTCTCTC
	Control (Actin2)	AtAct2_F1	TGGTGATGAAGCACAATCCAA
		AtAct2_R1	TGGACCAAGACTTCTGGGCAT

Table S2 Domains within NERD1		
DOMAIN	DOMAIN RANGE	ANALYTICAL TOOL (see Supplemental Methods for References)
Signal Peptide	1-21	SignalP 4.1 (1), PHOBIUS (2), InterPro Scan (3), TargetP (4)
Transmembrane	662-681	TMHMM (5), TMPred (6), PHOBIUS (2)
Cytoplasmic	682-698	TMHMM (5)
FG-GAP	54-78; 629-650	SSDB Motif (7), alignment with InterPro entry IPR013517
Alcohol Dehydrogenase-like	132-193; 238-274; 346-405	InterPro Scan (3)
Integrin-alpha-like	426-456; 564-652	InterPro Scan (3), Superfamily (8)

Table S2. Domains within NERD1 protein

In silico structural analysis of the NERD1 protein sequence was performed to explore its potential structure and function. Clearly identified in the protein are an N-terminal signaling peptide, a single transmembrane domain near the C-terminus, and a short C-terminal cytoplasmic domain. Ninety-five percent of the protein is predicted to be on the non-cytoplasmic side of the traversed membrane, but definitive functional domains of this portion of the protein were not identified. However, specific regions of the protein were found to have some similarity to domains found in integrins (i.e. an FG-GAP domain repeated multiple times in integrins, and a larger integrin-alpha domain), and some alcohol dehydrogenases. This similarity may, at least in part, be related to a common underlying tertiary structure that includes a beta propeller (see Figure S5).

	Roots examined	Root hairs evaluated	Root Hair Base Diameter (μm)		Maximum Shank Diameter (μm)		Percent Root Hair Rupture	
			Mean	Stdev	Mean	Stdev	Mean	Stdev
Col-0	18	203	16.4	3.0	14.5	2.2	1.8%	3.9%
exo84b-1	18	79	13.8	4.0	12.6	2.6	0.0%	0.0%
nerd1-1	18	200	21.5	6.7	26.4	9.1	26.3%	19.1%
nerd1-2	18	194	20.4	5.7	29.9	9.6	31.6%	17.6%
nerd1-3	18	196	20.0	5.2	30.2	10.0	33.9%	20.9%

Table S3. Morphology of *nerd1* root hairs compared to those of Col-0 and the *exo84b-1* severe exocyst mutant, when roots are growing within medium. Values highlighted in black are significantly different than those of Col-0 and *exo84b-1* ($p<0.001$, t-test).

Methods: *In silico* prediction of NERD1 tertiary structure, with References

Online threading programs [Swiss-Model (10), Phyre2 (11), pGenThreader (12), pDomThreader (12), Raptor-X (9), and ProFunc (13)] used the primary and predicted secondary structures of the NERD1 protein to identify similar template proteins for which the crystal structure has been solved and published. A hypothesized tertiary structure for NERD1 was thereby modeled from the known folds of the template proteins. These programs use different "threading" algorithms, report their threading alignments using different quality measures, and sometimes compare different sections of alignment between the query and template proteins. A summary of pertinent threading results is found in Data S2, including the range of NERD1 covered by each template comparison. Figure S4 (and Data S2 – Position of Beta-Propeller) shows a resultant model of NERD1 as derived by Raptor-X, which used 5 protein templates (4hdjA, 3p1lA, 4pk1A, 4cvbA, and 1yiqA) to arrive at its model. A similar model of NERD1's tertiary structure was obtained by Phyre2 (Data S2 – Position of Beta-Propeller) utilizing 10 templates (4cvcA, 1yiqA, 1tyea, 4v0nH, 4pk1A, 1kb0A, 4tqjA, 2c4dA, 1kv9A, and 4o9xA).

In general, it was found that the structure of NERD1 best aligned with proteins that had tertiary structures possessing a beta-propeller. As noted in the main text, beta propellers contain four to eight 3- or 4-stranded beta sheets arranged radially and pseudosymmetrically around a central axis (14). As shown (Figures S4) NERD1 is predicted by RaptorX to have seven beta sheets arranged in this manner. Beta propellers are widely used as structural scaffolds creating a surface for ligand binding and enzymatic activity (16). The beta-propeller-containing templates that best matched NERD1 in these threading analyses included: alcohol dehydrogenases and integrins, as might be expected from the domain analysis. Intriguingly, NERD1 is also predicted to share some structural similarity to portions of other proteins that interact with polysaccharides or glycoproteins, including lectins, carbohydrate binding proteins, and notably some pectin lyases and xyloglucanases.

Prediction of Calcium Binding Domains

The vast majority of proteins that showed some similarity to NERD1 in this threading analysis possessed at least one calcium binding domain (Data S2 – Threading Templates Table). In the RaptorX-modeled NERD1 tertiary structure a number of residues scattered throughout the protein were identified as the most likely ligand binding sites (Figure S4-C), forming a binding pocket for Ca++ (Figures S5-C and -D). In this Raptor-X model, loops extend from the two surfaces of the beta-propeller such that the protein extending from one surface of the propeller seems more extended and larger than that extending from the other surface (Figure S5-B). The calcium binding pocket is predicted to be located predominantly on this more extended side of the beta-propeller. RaptorX reports this predicted calcium binding pocket has a p-value of 2.48e-06 and a pocket multiplicity of 100. Pocket multiplicity is used to judge the quality of a predicted pocket, and represents the frequency with which the selected pocket was found in a set of ligand-binding protein structures. A multiplicity above 40 is considered a good indication that the predicted pocket is true.

Additional support for this calcium binding structure was sought using other *in silico* programs that analyzed ligand binding sites. 3DLigandSite (17) was used to assess for potential ligand binding sites in the NERD1 structural model predicted by Phyre2 (using 10 protein templates as noted above).

Additionally, pGenThreader was used to predict calcium binding sites in NERD1 using comparisons with single templates (2cn3, 4cag, 2zux, 2zuy, 4neh, 2bwr, 1flg, 1kb0, 1yiq, or 1kv9). Calcium binding sites in NERD1 were predicted by 3DLigandSite and pGenThreader, but the location of the predicted sites was not the same for each modeling program. Therefore, although calcium binding sites, and perhaps a calcium binding pocket, in NERD1 seems probable, the modeling results are not robust. The existence of a calcium binding pocket remains to be demonstrated, and its location, i.e. the residues involved, is currently speculative. Future research evaluating root growth sensitivity to various concentrations of extracellular calcium in *nerd1* mutants and controls would be informative.

<u>How does the predicted NERD1 Structure align with the highly-conserved regions of the protein?</u>

The eight regions of NERD1 highly conserved in Angiosperms (Data S1, Figure S3-A) were analyzed with respect to the putative structure of NERD1. Highly conserved region 1 coincides with one of the NERD1 FG-GAP domains, and region 7 encompasses most of the second FG-GAP domain. These regions also encompass portions of blades β-1 and β-2 in the predicted beta propeller. Overall, residues predicted to be components of the seven beta-sheet blades of the beta propeller (Figure S3) are more likely to fall within the highly conserved regions than would be predicted by chance ($p<0.001$, chi-square). Moreover, twenty of the 25 strands (i.e. 80%) forming the beta-propeller are found within the highly-conserved regions. This result is consistent with the hypothesis that this structural feature of the protein is important to its function, while suggesting that for some strands sequence identity across species is not required to maintain function.

Highly conserved region 2 is equivalent to the first of three sections of NERD1 identified as being an alcohol dehydrogenase-like domain. The other two sections of the alcohol dehydrogenase-like domain fall largely outside of those regions of NERD1 that are highly conserved in Angiosperms. One explanation for this inconsistency is that NERD1 and alcohol dehydrogenases share some structural similarity (e.g. a beta-propeller) rather than a functional similarity (e.g. the enzymatic oxidative role of dehydrogenases). In this regard, it is notable that the first two sections of the alcohol dehydrogenase-like domain encompass that part of NERD1 that includes all the beta strands forming two blades (β-2 and β-3 in Figure S3-D and Data S2) of NERD1's putative 7 bladed beta propeller.

Highly conserved region 8 coincides with the transmembrane domain of NERD1, and the short region (16 amino acids in Arabidopsis) found C-terminal to this domain is predicted to be cytoplasmic (Table S1). This C-terminal region varied in length in Angiosperms (15-19), Non-angiosperm Archaeplastida (17-40), and Metazoa (5-28), and showed a reduced degree of sequence conservation compared to the eight regions highly conserved in Angiosperms (Data S1). This suggests that this putatively cytoplasmic domain of the protein may not be central to its function. For example, the lack of a conserved cytoplasmic domain seems inconsistent with a hypothesis in which NERD1 function is modulated by an interaction of its cytoplasmic domain with a cytoplasmic signaling molecule.

SUPPLEMENTARY DATA REFERENCES

1. SignalP, http://www.cbs.dtu.dk/services/SignalP/
   Petersen, T.N., Brunak, S., Heijne, von, G. and Nielsen, H. (2011) SignalP 4.0: discriminating signal peptides from transmembrane regions. *Nat Meth*, **8**, 785–786.

2. PHOBIUS, http://phobius.sbc.su.se/
   Käll, L., Krogh, A. and Sonnhammer, E.L.L. (2007) Advantages of combined transmembrane topology and signal peptide prediction--the Phobius web server. *Nucleic Acids Res*, **35**, W429–32.

3. InterPro Scan, https://www.ebi.ac.uk/interpro/search/sequence-search
   Jones, P., Binns, D., Chang, H.-Y., et al. (2014) InterProScan 5: genome-scale protein function classification. *Bioinformatics*, **30**, 1236–1240.

4. TargetP, http://www.cbs.dtu.dk/services/TargetP/
   Emanuelsson, O., Nielsen, H., Brunak, S. and Heijne, von, G. (2000) Predicting subcellular localization of proteins based on their N-terminal amino acid sequence. *J Mol Biol*, **300**, 1005–1016.

5. TMHMM, http://www.cbs.dtu.dk/services/TMHMM/
   Möller, S., Croning, M.D. and Apweiler, R. (2001) Evaluation of methods for the prediction of membrane spanning regions. *Bioinformatics*, **17**, 646–653.

6. TMPred, http://embnet.vital-it.ch/software/TMPRED_form.html
   Hofmann, K. and Stoffel, W. (1993) TMbase - A database of membrane spanning proteins segments. Biol. Chem. Hoppe-Seyler **374**,166

7. SSDB Motif, http://www.genome.jp/tools/motif/
   Kanehisa, M., Goto, S., Kawashima, S. and Nakaya, A. (2002) The KEGG databases at GenomeNet. *Nucleic Acids Res*, **30**, 42–46.

8. Superfamily 1.75, http://supfam.org/SUPERFAMILY/
   Gough, J., Karplus, K., Hughey, R. and Chothia, C. (2001) Assignment of homology to genome sequences using a library of hidden Markov models that represent all proteins of known structure. *J Mol Biol*, **313**, 903–919.

9. RaptorX, http://raptorx.uchicago.edu/StructurePrediction/predict/
   Källberg, M., Wang, H., Wang, S., Peng, J., Wang, Z., Lu, H. and Xu, J. (2012) Template-based protein structure modeling using the RaptorX web server. *Nat Protoc*, **7**, 1511–1522.

10. Swiss-Model, https://swissmodel.expasy.org
Biasini, M., Bienert, S., Waterhouse, A., et al. (2014) SWISS-MODEL: modelling protein tertiary and quaternary structure using evolutionary information. *Nucleic Acids Res*, **42**, W252–8

11. Phyre2, http://www.sbg.bio.ic.ac.uk/~phyre2/html/page.cgi?id=index
Kelley, L.A., Mezulis, S., Yates, C.M., Wass, M.N. and Sternberg, M.J.E. (2015) The Phyre2 web portal for protein modeling, prediction and analysis. *Nat Protoc*, **10**, 845–858.

12. pGenThreader and pDomThreader, http://brylinski.cct.lsu.edu/content/pgenthreader
Lobley, A., Sadowski, M.I. and Jones, D.T. (2009) pGenTHREADER and pDomTHREADER: new methods for improved protein fold recognition and superfamily discrimination. *Bioinformatics*, **25**, 1761–1767.

13. ProFunc, http://www.ebi.ac.uk/thornton-srv/databases/ProFunc/
Laskowski, R.A., Watson, J.D. and Thornton, J.M. (2005) ProFunc: a server for predicting protein function from 3D structure. *Nucleic Acids Res*, **33**, W89–93.

14. **Springer, T.A.** (1997) Folding of the N-terminal, ligand-binding region of integrin alpha-subunits into a beta-propeller domain. *Proc Natl Acad Sci USA*, **94**, 65–72.

15. HHPred, https://toolkit.tuebingen.mpg.de/hhpred
Söding, J., Biegert, A. and Lupas, A.N. (2005) The HHpred interactive server for protein homology detection and structure prediction. *Nucleic Acids Res*, **33**, W244–8.

16. **Kopec, K.O. and Lupas, A.N.** (2013) β-Propeller blades as ancestral peptides in protein evolution. *PLoS ONE*, **8**, e77074.

17. 3DLigandSite, http://www.sbg.bio.ic.ac.uk/3dligandsite/
Wass, M.N., Kelley, L.A. and Sternberg, M.J.E. (2010) 3DLigandSite: predicting ligand-binding sites using similar structures. *Nucleic Acids Res*, **38**, W469–73.

18. PredictProtein, https://www.predictprotein.org/
Yachdav, G., Kloppmann, E., Kajan, L., et al. (2014) PredictProtein--an open resource for online prediction of protein structural and functional features. *Nucleic Acids Res*, **42**, W337–43.

19. PDBSum, http://www.ebi.ac.uk/thornton-srv/databases/cgi-bin/pdbsum/GetPage.pl?pdbcode=index.html
Laskowski, R.A., Hutchinson, E.G., Michie, A.D., Wallace, A.C., Jones, M.L. and Thornton, J.M. (1997) PDBsum: a Web-based database of summaries and analyses of all PDB structures. *Trends Biochem Sci*, **22**, 488–490.

20. PsiPred, http://bioinf.cs.ucl.ac.uk/psipred/
Jones, D.T. (1999) Protein secondary structure prediction based on position-specific scoring matrices. *J Mol Biol*, **292**, 195–202.
